# Context-dependent induction of autoimmunity by TNF signaling deficiency

**DOI:** 10.1172/jci.insight.149094

**Published:** 2022-03-08

**Authors:** Tam D. Quach, Weiqing Huang, Ranjit Sahu, Catherine M.M. Diadhiou, Chirag Raparia, Roshawn Johnson, Tung Ming Leung, Susan Malkiel, Peta Gay Ricketts, Stefania Gallucci, Çagla Tükel, Chaim O. Jacob, Martin L. Lesser, Yong-Rui Zou, Anne Davidson

**Affiliations:** 1 Institute of Molecular Medicine, Feinstein Institutes for Medical Research, Manhasset, New York, New York, USA.; 2 Donald and Barbara Zucker School of Medicine at Hofstra/Northwell and; 3 Biostatistics Unit, Feinstein Institutes for Medical Research, Manhasset, New York, New York, USA.; 4 Department of Microbiology, Immunology and Inflammation, Lewis Katz School of Medicine, Temple University, Philadelphia, Pennsylvania, USA.; 5 Division of Rheumatology, Department of Medicine, Keck School of Medicine, University of Southern California, Los Angeles, California, USA.

**Keywords:** Immunology, Autoimmune diseases

## Abstract

TNF inhibitors are widely used to treat inflammatory diseases; however, 30%–50% of treated patients develop new autoantibodies, and 0.5%–1% develop secondary autoimmune diseases, including lupus. TNF is required for formation of germinal centers (GCs), the site where high-affinity autoantibodies are often made. We found that TNF deficiency in Sle1 mice induced TH17 T cells and enhanced the production of germline encoded, T-dependent IgG anti-cardiolipin antibodies but did not induce GC formation or precipitate clinical disease. We then asked whether a second hit could restore GC formation or induce pathogenic autoimmunity in TNF-deficient mice. By using a range of immune stimuli, we found that somatically mutated autoantibodies and clinical disease can arise in the setting of TNF deficiency via extrafollicular pathways or via atypical GC-like pathways. This breach of tolerance may be due to defects in regulatory signals that modulate the negative selection of pathogenic autoreactive B cells.

## Introduction

TNF is a potent inflammatory cytokine with pleiotropic effects on both cell survival and death. TNF has 2 receptors. TNFR1 has an intracellular death domain, is ubiquitously expressed, and preferentially binds soluble TNF with high affinity (1–3). Signaling via TNFR1 induces inflammatory gene expression but also can induce apoptosis via a caspase-3–dependent pathway to dampen immune activation (3). TNFR2 lacks an intracellular death domain, is expressed mostly by lymphocytes, and binds membrane-bound TNF. TNFR2 signaling induces T cell activation and proliferation (4) and can costimulate B cells (5). There may also be an antiinflammatory role for TNFR2 since a specific TNFR2 agonist can induce Tregs and induce apoptosis of autoreactive CD8^+^ T cells (6, 7).

Although TNFR1 is classically thought to be the main receptor involved in TNF-mediated tissue injury, this is not universally true. In acute experimental immune complex–mediated glomerulonephritis, TNFR1 deficiency improved disease initially, but late exacerbation occurred due to failed deletion of activated T cells. Renal TNFR2 expression was also critical for the effector component of disease by regulating interstitial macrophage accumulation (8). Similarly, dependence of vascular inflammation on TNFR2 was found in mice that overexpress TNF and develop spontaneous CNS pathology (9). Nevertheless, the loss of TNFR2 on nonhematopoietic cells increases chronic inflammation in experimental autoimmune encephalitis due to a decrease in Tregs (10).

Paradoxically, both TNF excess (11–15) and deficiency (16, 17) can exacerbate autoimmunity. Findings that excess TNF promotes inflammation led to the development of TNF inhibition as a first-line biologic therapy for many autoimmune diseases, including rheumatoid arthritis, psoriasis, and inflammatory bowel disease. By contrast, although anti-TNF therapy can induce remission of refractory nephritis (18, 19), TNF deficiency is associated with induction of systemic lupus erythematosus (SLE) in more than one mouse model (15, 20, 21). A role for TNF deficiency in the induction of autoimmunity in humans is supported by the finding that up to 50% of patients with autoimmune disorders treated with TNF inhibitors develop de novo anti-nuclear antibodies (ANAs), 15% develop antibodies against DNA and/or cardiolipin, and 0.2%–1% develop clinical SLE that often remits upon cessation of treatment (22–24). TNF inhibitors can also induce multiple sclerosis–like syndromes (25, 26), psoriasis (27), and vasculitis (28, 29). Considering the number of patients who are treated with TNF inhibitors worldwide, secondary autoimmune disease development is a significant medical problem.

TNF inhibition affects B cell homeostasis and selection via disorganization of the B cell follicle and disruption of the follicular dendritic cell (FDC) network (30, 31). This is due to the absence of the interaction of TNFR1 on the FDCs with soluble TNF provided by B cells (30, 32, 33) and is associated with decreased expression of CXCL13 that compromises the recruitment of CXCR5-expressing B cells and T follicular helper (TFH) cells to germinal centers (GCs). Loss of GCs occurs in both mice and humans treated with TNF inhibitors (33, 34).

Proper GC function and organization are pivotal for both negative and positive selection of B cells, allowing them to rewire their activation and regulatory signals to balance between cell proliferation and death while promoting clonal selection, as well as memory and plasma cell differentiation (35). In addition, TNF-TNFR1 interactions dampen immune responses by triggering activation-induced cell death (AICD) of T cells (36). Thus, it is not altogether surprising that TNF inhibition may induce a breach of B cell tolerance. Nevertheless, the origins of the autoantibodies that arise in patients treated with TNF inhibitors and the reason why only a few of these patients develop clinical lupus have not been fully explored. In this study, we used mouse models of SLE in which autoimmunity is altered by the absence of TNF and explored the contribution of secondary immune insults to the induction of clinical SLE.

## Results

### Loss of TNF or TNF receptors does not decrease survival of Sle1 mice.

**Figure 1:**
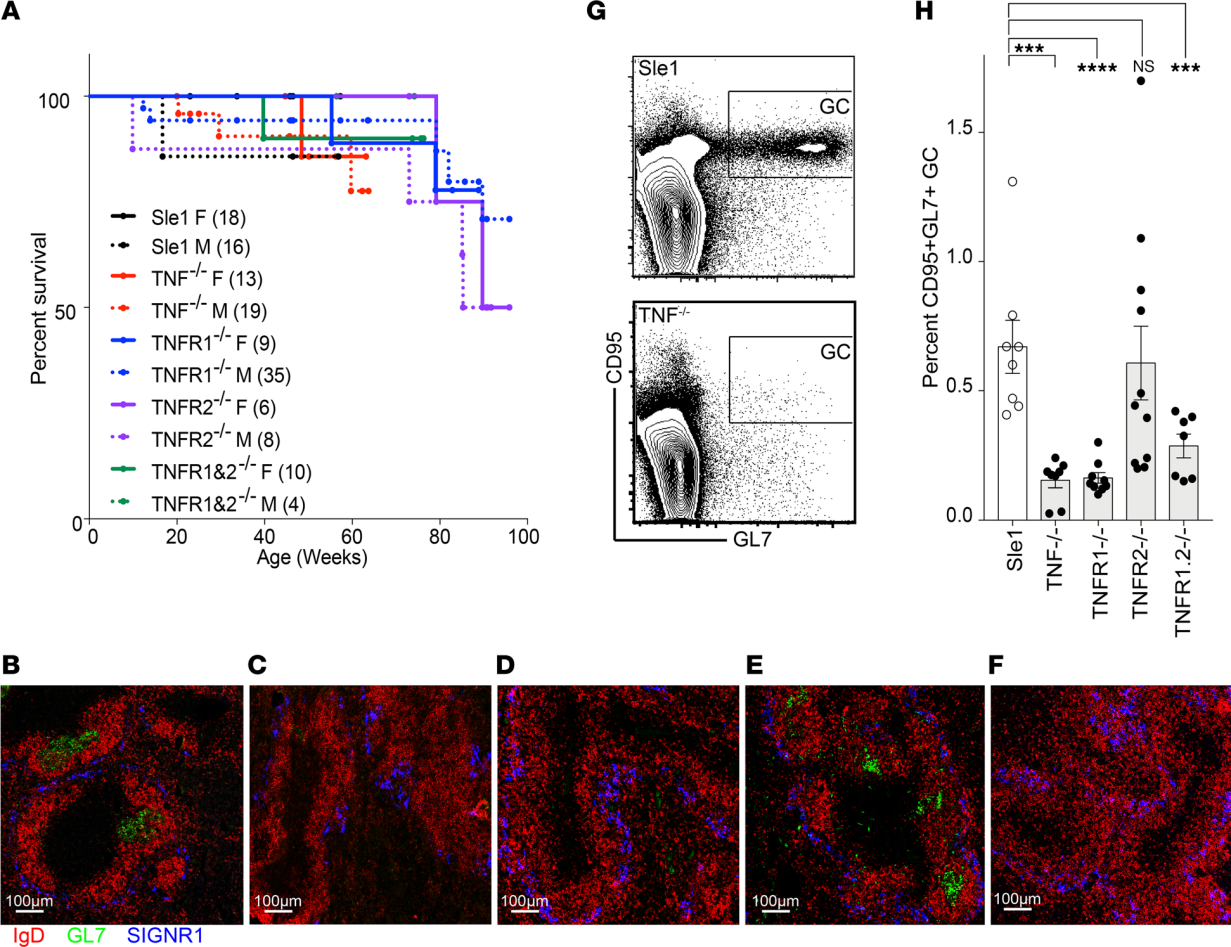
TNF signaling via TNFR1 is required for GC formation. (**A**) Survival curves of female and male Sle1 mice of the indicated genotype. Numbers of mice per group are indicated in parentheses. (**B**–**F**) GC staining of >6-month-old Sle1 (**B**), Sle1 TNF^–/–^ (**C**), Sle1 TNFR1^–/–^ (**D**), Sle1 TNFR2^–/–^ (**E**), and Sle1 TNFR1&2^–/–^ (**F**) mice (original magnification, 10×), representative of 3–5 mice per group. (**G**) Flow plots show gating of splenic GC (CD95^+^GL7^+^) cells in CD19^+^ B cells from >9-month-old Sle1 (top) and Sle1 TNF^–/–^ mice (bottom). (**H**) Bar graph shows the summary results of **G**. Dots on bar graphs represent individual mice. ANOVA Kruskal-Wallis with Dunn’s multiple comparisons test, *P < 0.05: **P < 0.01, ***P < 0.001, ****P < 0.0001. SIGNR1, CD209b.

Aged Sle1 mice develop autoantibodies to chromatin and cardiolipin (CL) but do not develop glomerulonephritis unless a second genetic defect is introduced (37). Deficiency of TNF, TNFR1, TNFR2, or both TNFR1&2 did not precipitate lupus nephritis in either male or female Sle1 mice as manifested by absence of proteinuria >30 mg/dL and/or premature mortality in mice aged to >1 year (Figure 1A).

### Loss of TNF or TNFR1 alters splenic immune effector cell phenotype.

Few differences were observed between strains in the frequency or total number of B cells or B cell subsets and CD4^+^ T cells as the mice aged (Supplemental Figure 1, A–H; supplemental material available online with this article; https://doi.org/10.1172/jci.insight.149094DS1). Sle1 and Sle1.TNFR2^–/–^ mice formed spontaneous GCs by 6 months of age, but these were absent in Sle1.TNF^–/–^, TNFR1^–/–^, and TNFR1&2 double deficient mice (Figure 1, B–H), with no differences between males and females. Formation of FDC clusters and GCs requires TNFR1 expression on FDCs and TNF expression on B cells (38). Accordingly, FDCs could not be identified in Sle1.TNF^–/–^ or TNFR1^–/–^ mice, and the morphology of the follicles was abnormal, with the accumulation of a ring of CD21/CD35^+^ cells in the B cell zone (Supplemental Figure 2A). By using bone marrow chimeras in which bone marrow cells from Sle1 mice were transplanted into lethally irradiated Sle1.TNFR1^–/–^ mice, we showed that contrary to what has previously been reported, these CD21/35^+^ cells are CD45^+^ B cells and not stromal FDCs (Supplemental Figure 2B).

**Figure 2:**
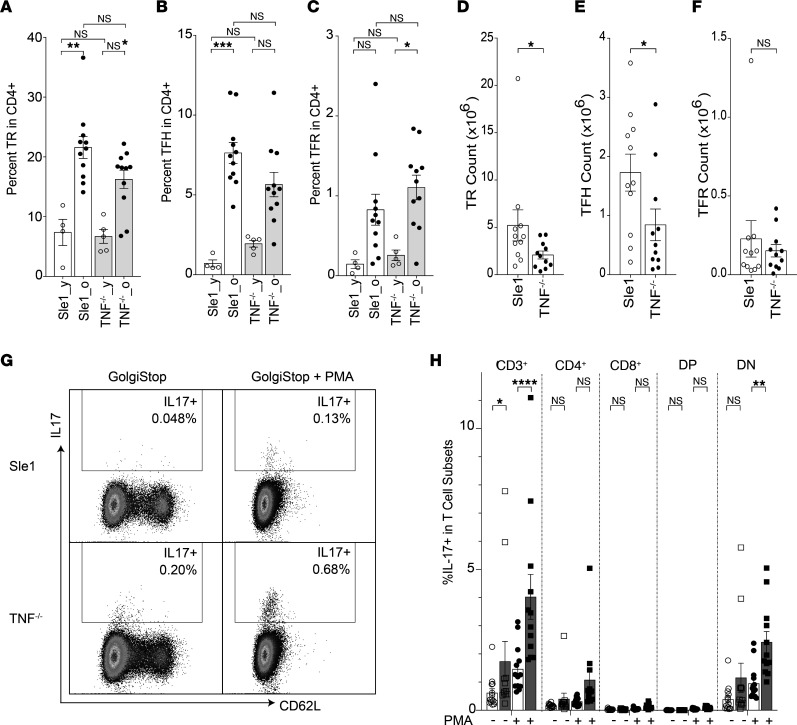
Inflammatory effector T cells are enhanced in TNF-deficient mice. (**A**–**C**) Bar graphs show the percentage of Tregs (TR — CD3^+^CD4^+^Foxp3^+^) (**A**), TFH cells (CD3^+^CD4^+^PSGL1^–^CD44^+^PD1^hi^Bcl6^hi^) (**B**), and TFR cells (CD3^+^CD4^+^PSGL1^–^CD44^+^PD1^hi^Bcl6^hi^Foxp3^+^) (**C**) in CD4^+^ T cells of Sle1 and Sle1 TNF^–/–^ mice (y, young, 2–3 months old; o, old, >8 months old). ANOVA Kruskal-Wallis with Dunn’s multiple comparisons test, *P < 0.05: **P < 0.01, ***P < 0.001. (**D**–**F**) Summary results of TR, TFH, and TFR cell counts in old mice, Mann-Whitney nonparametric test, *P < 0.05. (**G**) Plots show IL-17 expression in CD4^+^ T cells with (+)/without (-) PMA stimulation from Sle1 and Sle1 TNF^–/–^ mice. (**H**) Summary bar plot shows the percentage of IL-17^+^ T cells in total CD3^+^ and indicated T cell subsets with/without PMA stimulation. Sle1 mice are shown in unfilled symbols, and Sle1 TNF^–/–^ mice are shown in filled symbols. Ordinary 2-way ANOVA with Tukey’s multiple comparisons test, *P < 0.05: **P < 0.01, ****P < 0.0001. DP, CD4^+^CD8^+^; DN, CD4^–^CD8^–^.

Although GCs were absent in Sle1.TNF^–/–^ and Sle.TNFR1^–/–^ mice, we detected an increased frequency of programmed cell death 1–high, selectin P ligand–negative (PD1^hi^PSGL1^–^) CD4^+^ T cells as the mice aged (Supplemental Figure 1I). Further flow cytometric analysis showed that the frequency of effector TFH cells (PD1^hi^Bcl6^hi^FoxP3^–^), T follicular regulatory cells (TFR cells) (PD1^hi^Bcl6^hi^FoxP3^+^), and Tregs (CD4^+^Foxp3^+^) did not differ between aged Sle1 and Sle1 TNF^–/–^ mice (Figure 2, A–C, and Table 1), although their total count was modestly lower than in Sle1 controls (Figure 2, D–F, and Table 1). In Sle1 mice, TFH and TFR cells were located within GCs, and Bcl6 was found within both B cells and CD4^+^ T cells in the GC (Supplemental Figure 3A). By contrast, PD1^+^ and Bcl6^+^ CD4^+^ T cells and FoxP3^+^CD4^+^ T cells were scattered within the T cell zone of Sle1 TNF^–/–^ mice (Supplemental Figure 3, A and B). It has previously been reported that TNF deficiency in SLE-prone NZM2328 (NZM) mice results in the accumulation of TH17 cells (39). We similarly found a significantly increased frequency of IL-17–producing T cells, with double-negative CD8^–^CD4^–^ cells being the predominant reservoir (Figure 2, G and H, and Table 1).

### Loss of TNF or TNF receptors alters the autoantibody profile and plasma cell repertoire of Sle1 mice.

**Figure 3:**
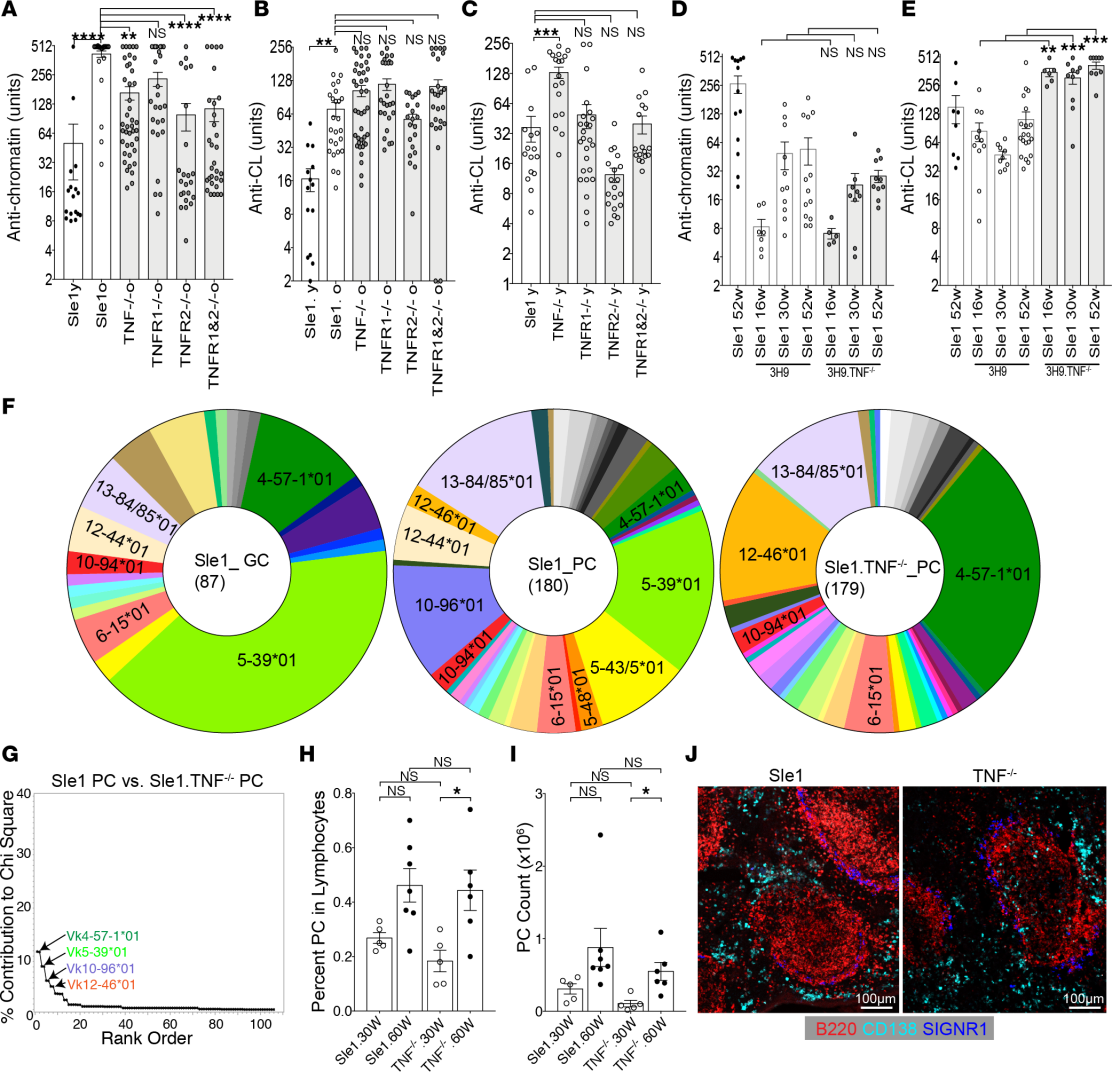
Altered autoantibody specificity in Sle1 TNF^–/–^ and Sle1 TNFR1^–/–^ mice. (**A** and **B**) Bar graphs show the relative units of IgG antibodies against chromatin (**A**) and CL/β2GP1 (**B**) from sera of >9-month-old Sle1 mice of the indicated genotypes. (**C**) Bar graphs show the relative units of IgG antibodies against CL/β2GP1 from sera of 2- to 3-month-old Sle1 mice of the indicated genotypes. (**D** and **E**) Bar graphs show IgG antibodies against chromatin (**D**) and CL (**E**) from sera of 3H9 Sle1 and 3H9 Sle1 TNF^–/–^ mice at sequential ages. (**F**) Pie charts show percentage of Igk light chain V region (Vk) gene usage in 3H9^+^ GC cells and plasma cells (PCs) from >9-month-old 3H9 Sle1 and PCs from 3H9 Sle1 TNF^–/–^ mice. Fisher’s exact test rows × columns table, *P* < 0.0005. (**G**) Scree plot shows the percentage contribution to the χ^2^ analysis of the most overrepresented Vk genes in PCs of 3H9 Sle1 and 3H9 Sle1 TNF^–/–^ mice. (**H** and **I**) Percentage (**H**) and number (**I**) of 3H9^+^ PCs from Sle1 and Sle1 TNF^–/–^ mice at sequential ages. (**J**) Spleen CD138^+^ PCs are present in the extrafollicular region in >9-month-old Sle1 and Sle1 TNF^–/–^ mice, representative of 3–5 mice per group. Dots on bar graphs represent individual mice. ANOVA Kruskal-Wallis with Dunn’s multiple comparisons test, *P < 0.05: **P < 0.01, ***P < 0.001, ****P < 0.0001. y, young (2–3 months); o, old (>9 months); w, weeks.

Sle1 mice developed autoantibodies against chromatin and CL/beta2 glycoprotein1 (β2GP1) as they aged (Figure 3 and Supplemental Figure 4, A–C). Sle1 TNF^–/–^ and Sle1 TNFR1&2 double deficient mice of both sexes had significantly lower titers of anti-chromatin autoantibodies than Sle1 mice (Figure 3A). Sle1 TNF^–/–^ mice developed anti-cardiolipin antibodies at a younger age than Sle1 mice despite the absence of GCs (Figure 3B). This difference was no longer significant in aged mice (Figure 3C). Sle1 TNFR2^–/–^ mice had significantly lower titers of anti-chromatin and anti-dsDNA antibodies compared with Sle1 mice (Figure 3A and Supplemental Figure 4D) despite the presence of spontaneous GCs, consistent with the reported role of TNFR2 as a B cell costimulatory molecule (5).

Further mechanistic experiments were performed using Sle1 TNF^–/–^ mice in which there was a preference for anti-cardiolipin specificity over anti-chromatin specificity. To determine the mechanism for the change in tolerance to autoantigens in these mice, we introduced the autoreactive heavy chain gene 3H9 that confers anti-DNA, anti-chromatin, and/or anti-cardiolipin activity depending on the associated Vk gene (40–42). In this transgenic model, anti-chromatin antibody titers were similar in 3H9 Sle1 and 3H9 Sle1 TNF^–/–^ mice (Figure 3D) whereas anti-cardiolipin antibodies were strongly induced in 3H9 Sle1 TNF^–/–^ mice, irrespective of age (Figure 3E).

To determine the source of autoreactive B cells in 3H9 Sle1 TNF^–/–^ mice, we analyzed the repertoire of light chains associated with the 3H9 heavy chain in sorted B cell subsets from 3H9 Sle1 and 3H9 Sle1 TNF^–/–^ mice using single-cell PCR. Approximately 60% of all Vk light chains that associate with the 3H9 heavy chain confer autoreactivity with lupus-associated autoantigens (40–42). We have previously established that reconstituted IgG antibodies with unmutated 3H9/Vk combinations using Vk1-117, 3-4, 3-12, 4-55, 9-120, 13-85, and 16-104 are nonreactive with dsDNA, CL, histones, or chromatin whereas those using Vk1-110, 4-57, 4-57*1-01, 4-79, 5-43, 5-45, 5-48, 6-15, 8-30, 9-123, 10-94, and 12-46 react with 1 or more of these antigens (43). Furthermore, in other lupus-prone strains, the 3H9-associated GC and PC repertoires are dominated by members of the Vk5 family that are infrequently expressed in marginal zone or follicular B cells (44).

The follicular 3H9-associated Vk repertoire in 3H9 Sle1 and 3H9 Sle1 TNF^–/–^ mice was diverse, with modest differences observed between the 2 strains (Supplemental Figure 4, E and G). Vk3-12 was overrepresented in the Sle1 strain, a finding of unclear significance since this light chain does not confer autoreactivity when associated with the 3H9 heavy chain and was infrequently detected among PCs. Similarly, Vk2-112 was overrepresented in the Sle1 TNF^–/–^ strain but was not present in the PC repertoire. The marginal zone repertoire in 3H9 Sle1 and 3H9 Sle1 TNF^–/–^ mice was highly restricted, with a high frequency of Vk12-46 light chains as we have previously reported in other lupus strains (Supplemental Figure 4, F and H) (43, 45). We next examined the GC repertoire in 3H9 Sle1 mice, and because GCs are not present in Sle1 TNF^–/–^ mice, we also examined the PC repertoire in both strains. Five of 10 aged 3H9 Sle1 mice with high-titer autoantibodies manifested a high frequency of Vk5 gene family members in the GC and PC compartments. Members of this family confer anti-chromatin, anti-dsDNA, and anti-cardiolipin activity when associated with the 3H9 heavy chain (43). By contrast, Vk5 light chains were infrequent in the PC compartment of 3H9 Sle1 TNF^–/–^ mice, reflecting the absence of GCs in which these antibodies are generated; instead PCs from these mice used a high frequency of Vk4-57-1*01 and Vk12-46, light chains that are associated with anti-cardiolipin autoreactivity (43) (Figure 3, F and G). These findings are consistent with the serologic alterations in the Sle1 TNF^–/–^ mice. To determine whether the light chains from 3H9-encoded Sle1 TNF^–/–^ PCs had undergone somatic mutation, we generated a primer for the Vk4-57*1-01 leader sequence and sequenced the full-length light chain from 20 single PCs. All 20 light chains were germline encoded. Together, these findings show that whereas the anti-chromatin response is positively selected in GCs of Sle1 mice, the anti-cardiolipin response in TNF-deficient mice is generated outside GCs. PCs were present in equal number in Sle1 and Sle1 TNF^–/–^ spleens and located in extrafollicular sites in both strains (Figure 3, H–J).

### Anti-cardiolipin autoantibody production in TNF-deficient mice is CD4^+^ T cell dependent.

**Figure 4:**
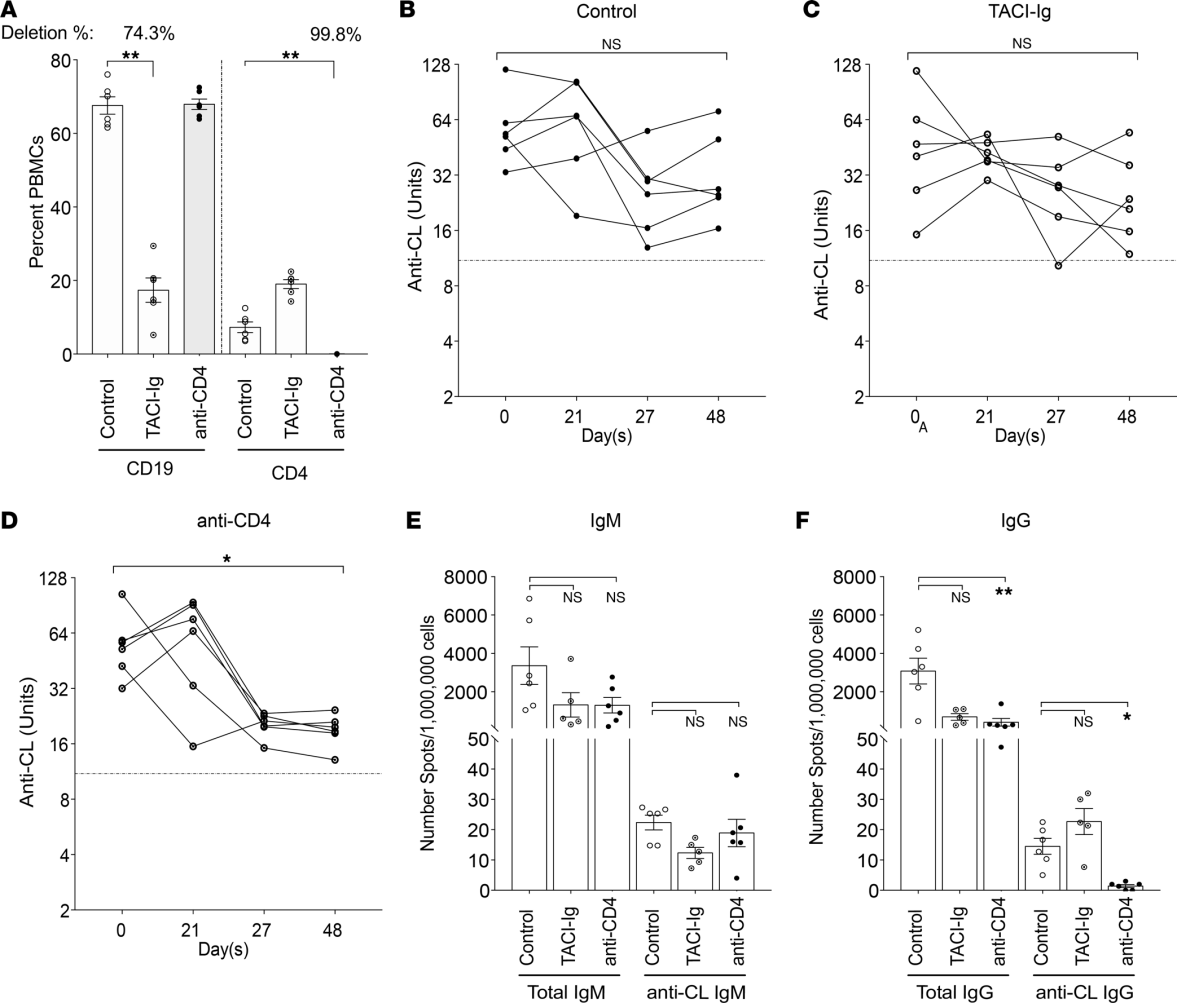
IgG anti-cardiolipin production in Sle1 TNF^–/–^ mice is CD4 dependent. (**A**) Percentage of CD19^+^ and CD4^+^ in total PBMCs from Sle1 TNF^–/–^ mice after TACI-Ig or anti-CD4 treatment, Mann-Whitney nonparametric test, **P < 0.005. (**B**–**D**) Changes in titers of serum IgG anti-cardiolipin antibodies in untreated (**B**) and TACI-Ig– (**C**) and anti-CD4– (**D**) treated mice. Dotted line represents the mean value for 5 C57BL/6 mice >9 months old. Repeated measures ANOVA Kruskal-Wallis, *P < 0.05. (**E** and **F**) ELISPOT assay shows number of spleen cells secreting IgM (**E**) and IgG (**F**) and total Ig and anti-cardiolipin antibodies. ANOVA Kruskal-Wallis with Dunn’s multiple comparisons test, *P < 0.05, **P < 0.005.

We next asked whether the generation of the extrafollicular anti-cardiolipin response in Sle1 TNF^–/–^ mice requires either CD4^+^ T cells or B cell activating factor (BAFF) by treating 5- to 6-month-old mice with either anti-CD4 or transmembrane activator and CAML interactor–Ig (TACI-Ig) for 8 weeks. As expected, CD19^+^ B cells were depleted by approximately 74% in TACI-Ig–treated mice whereas CD4^+^ T cells were depleted by more than 99% in anti-CD4–treated mice (Figure 4A). Serum levels of anti-cardiolipin IgG antibody were significantly reduced after treatment with anti-CD4 compared with before treatment whereas serum anti-cardiolipin IgG antibody levels were not significantly different after TACI-Ig treatment or no treatment (Figure 4, B–D). However, there were no differences when treatment groups were compared with the control group. We therefore used ELISPOT assay to determine the frequency of antibody-secreting cells at the end of the experiment. By week 8 after treatment, the frequency of total IgM-producing cells was not significantly different between the treated mice and untreated controls. The frequency of total IgG-secreting cells was significantly reduced in both treated groups compared with controls; however, the frequency of IgG anti-cardiolipin–producing B cells was significantly decreased only in the anti-CD4–treated group (Figure 4, E and F). These data show that the extrafollicular IgG anti-cardiolipin response in Sle1 TNF^–/–^ mice is CD4^+^ T cell dependent but BAFF independent.

### Innate immune stimulus induces lupus-associated autoantibody production in TNF-deficient mice.

**Figure 5:**
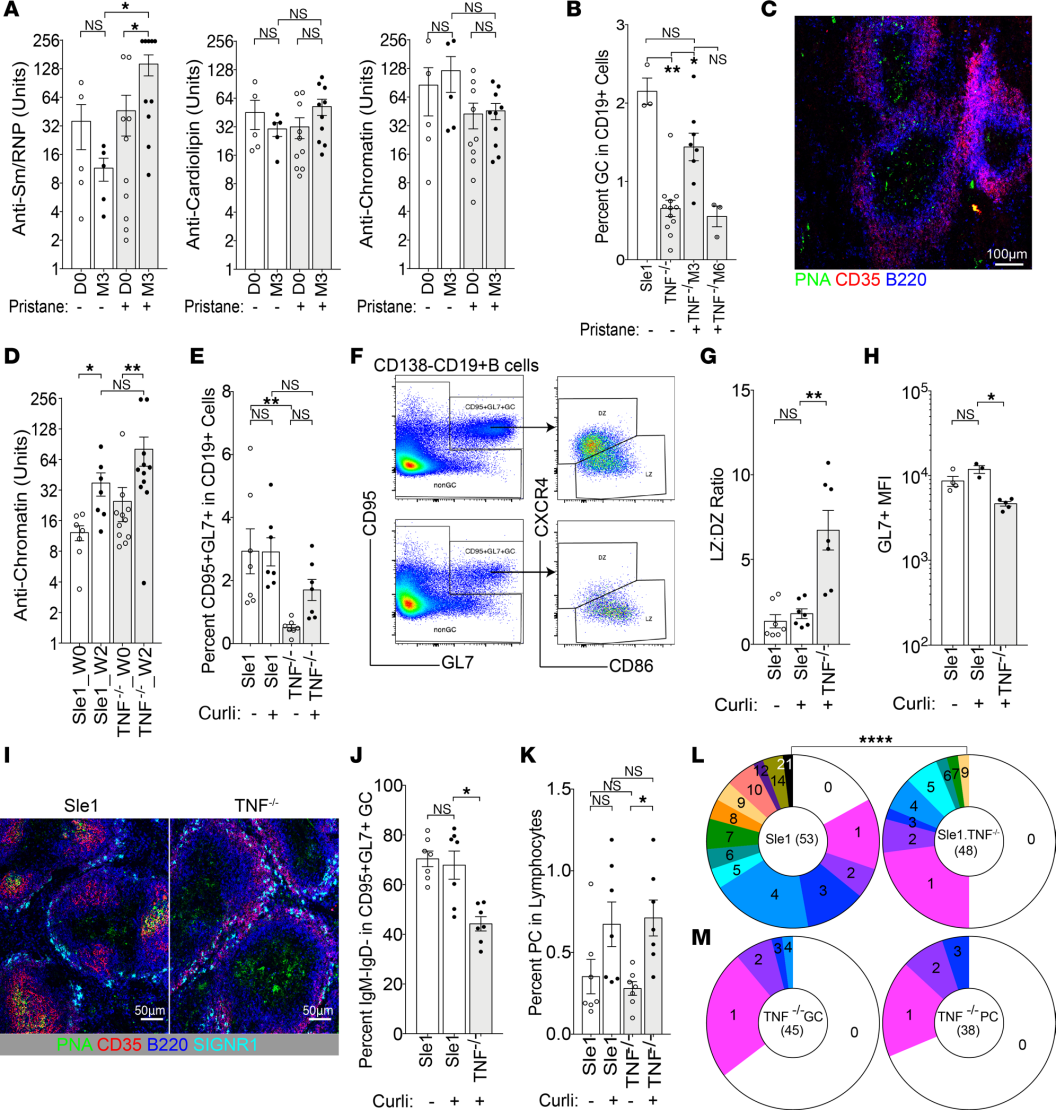
Innate stimulus induces activated GL7^+^ B cells and lupus-associated autoantibody production in Sle1 TNF^–/–^ mice. (**A**) Bar graphs show the relative units of IgG antibodies against Sm/RNP, CL, and chromatin (left to right) from sera of 12-week-old mice at day 0 (D0) and 3 months after (M3) pristane treatment. (**B**) Plot shows percentage of CD95^+^GL7^+^ cells in CD19^+^ B cells from pristane-treated mice. (**C**) Scattered PNA^+^ non–B cells are present in the T cell zone in Sle1 TNF^–/–^ mice, 3 months after pristane treatment (original magnification, 10×). (**D**) Plot shows the relative units of IgG antibodies against chromatin in curli-treated mice. (**E**) Percentage of CD95^+^GL7^+^ cells from curli-treated mice. (**F**) Flow plots show the gating of splenic CD95^+^GL7^+^ B cells and their respective light zone (LZ) and dark zone (DZ) phenotype from Sle1 (top) and Sle1 TNF^–/–^ mice (bottom). (**G** and **H**) Bar graphs show the summary result of LZ/DZ ratio (**G**) and GL7 expression (**H**). (**I**) PNA^+^ B cells are present in GCs in Sle1 mice and in the T cell zone of Sle1 TNF^–/–^ mice, 6 weeks after curli treatment (original magnification, 20×). (**J** and **K**) Bar graph shows the percentage of IgM^–^IgD^–^ cells in CD95^+^GL7^+^ B cells (**J**) and percentage of IgD^–^CD138^+^ PCs in total live lymphocytes (**K**). (**L**) Pie charts show percentage of VH sequences with mutations from single CD95^+^GL7^+^ B cells in curli-treated Sle1 (left) and Sle1 TNF^–/–^ (right) mice. Each slice represents the proportion of cells with the indicated number of mutations. χ^2^ analysis, ****P < 0.0001. Dots on bar graphs represent individual mice. (**M**) Pie charts show percentage of IGKV4-57-1*01 sequences with mutations from single CD95^+^GL7^+^ GCs (left) and CD138^+^ PCs (right) B cells in curli-treated 3H9 Sle1 TNF^–/–^ mice. Each slice represents the proportion of cells with the indicated number of mutations. (**A**, **B**, **D**, **E**, **G**, **H**, **J**, and **K**) ANOVA Kruskal-Wallis with Dunn’s multiple comparisons test, *P < 0.05, **P < 0.01. Dots on bar graphs represent individual mice. Immunohistochemistry represents 3–5 mice per group.

Our data so far show loss of GCs and induction of an extrafollicular, germline encoded anti-cardiolipin response in TNF-deficient Sle1 mice. This finding is reminiscent of the nonpathogenic autoreactivity induced in approximately 30% of patients treated with TNF inhibitors. We next asked whether pathogenic autoimmunity, as observed in only a small percentage of treated patients, could be induced by an exogenous stimulus. We first tested several different innate stimuli. Pristane has previously been reported to induce autoimmunity and anti–Smith and ribonucleoprotein antigens (anti-Sm/RNP) autoantibodies in C57BL/6 TNF^–/–^ mice by a type 1 IFN–dependent mechanism (21). We found that pristane similarly induced an increase in anti-Sm/RNP autoantibodies in Sle1 TNF^–/–^ mice without any changes in anti-cardiolipin and anti-DNA specificities (Figure 5A). However, pristane did not induce proteinuria or shorten the life span of Sle1 TNF^–/–^ mice. CD95^+^GL7^+^ B cells were briefly elevated at 3 months in Sle1 TNF^–/–^ mice but waned by 6 months after pristane treatment (Figure 5B). GC structures were not observed in these mice by immunohistochemistry either at 3 or at 6 months, with only a few scattered peanut agglutinin^+^ (PNA^+^) cells visualized within the T cell region of the B cell follicle (Figure 5C). These data show that pristane induces an enhanced anti-Sm/RNP response in Sle1 TNF^–/–^ mice without the formation of GCs and without induction of clinical disease.

Antibodies against the bacterial DNA–binding amyloid protein curli correlate with flares in patients with lupus, suggesting that bacterial curli can be a disease trigger (46). This protein induces anti-DNA antibodies in both non-autoimmune mice and autoimmune mice in a TLR2- and TLR9-dependent fashion (47, 48). We found that curli treatment induced anti-chromatin antibodies in both Sle1 and Sle1 TNF^–/–^ mice (Figure 5D). Although the frequency of CD95^+^GL7^+^ GC B cells did not increase in Sle1 mice after curli treatment, CD95^+^GL7^+^PNA^+^ B cells were increased in Sle1 TNF^–/–^ mice 6 weeks after curli treatment compared with untreated Sle1 TNF^–/–^ controls, reaching the same frequency as in Sle1 mice (Figure 5E). However, these cells were predominantly of the light zone (LZ) (CXCR4^–^CD86^+^) phenotype (Figure 5, F and G), and their level of GL7 expression was significantly lower than that of curli-treated Sle1 controls (Figure 5H). Furthermore, these cells did not cluster into a GC structure but were scattered diffusely within the T cell zone (Figure 5I). Class switching was also impaired in these cells, with a lower percentage of class-switched cells compared with those from their Sle1 counterparts (Figure 5J). An increase in PCs was found both in curli-treated Sle1 TNF^–/–^ and in curli-treated Sle1 mice (Figure 5K). Analysis of immunoglobulin heavy chain variable region (VH) gene sequences from single CD95^+^GL7^+^PNA^+^ cells from curli-treated Sle1 TNF^–/–^ mice showed significantly less somatic hypermutation compared with those of curli-treated Sle1 controls (Figure 5L). Together, these data show that the autoantibody response in curli-treated Sle1 TNF^–/–^ mice is associated with the induction of atypical, activated B cells that are located predominantly within the T cell zones and have low levels of somatic mutation.

To determine whether there was a change in selection of autoreactive B cells upon curli administration, we treated 2- to 3-month-old 3H9 Sle1 and 3H9 Sle1 TNF^–/–^ mice with curli and analyzed the 3H9-associated Vk repertoire 6 weeks later. Although curli treatment induced atypical CD95^+^GL7^+^ B cells in 3H9 Sle1 TNF^–/–^ mice as it did in nontransgenic Sle1 TNF^–/–^ mice, it enhanced only the anti-cardiolipin response in both strains of 3H9 transgenic mice, with little induction of anti-chromatin or anti-DNA antibodies. Analysis of the 3H9-associated Vk repertoire of CD95^+^GL7^+^PNA^+^ B cells and PCs from curli-treated 3H9 Sle1 TNF^–/–^ mice showed a high frequency of Vk4-57*1-01 light chains, similar to that found in their untreated counterparts (66% and 44% of the repertoire, respectively). However, mutation analysis of full-length Vk4-57*1-01 light chains from both these B cell subsets now showed a similar frequency of somatic mutations as in Figure 5L, with 33%–35% of the cells harboring at least 1 mutation (Figure 5M).

**Figure 6:**
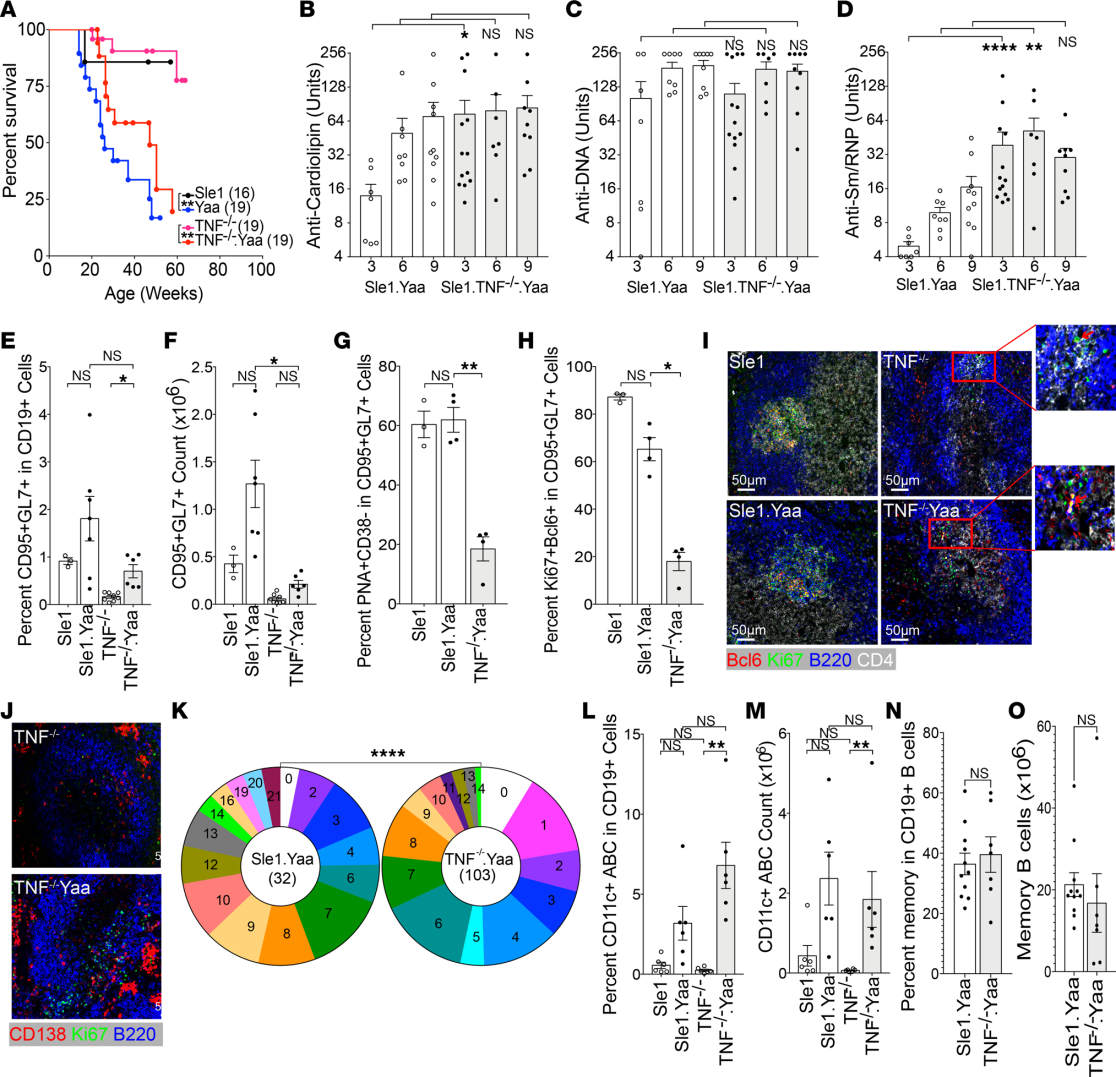
TLR7 overexpression induces pathogenic autoantibodies in Sle1 TNF^–/–^ mice. (**A**) Survival plots of male Sle1 mice of the indicated genotypes. Log-rank test **P < 0.01. (**B**–**D**) Plots show relative units of IgG antibodies against cardiolipin (**B**), DNA (**C**), and Sm/RNP (**D**) from sera of male Sle1 mice of the indicated genotype at 3, 6, and 9 months of age. (**E**–**H**) Summary bar graphs of flow cytometry analysis. (**E** and **F**) Percentage and count of CD95^+^GL7^+^ cells in CD19^+^ B cells from male Sle1, Sle1 Yaa, Sle1 TNF^–/–^, and Sle1 Yaa TNF^–/–^ mice. (**G** and **H**) Percentage of PNA^+^CD38^–^ (**G**) and Ki67^+^Bcl6^+^ (**H**) cells in splenic CD95^+^GL7^+^ B cells. (**I**) Immunohistochemistry images (original magnification, 20×) show Ki67^+^Bcl6^+^ B cells in GCs of male Sle1 (top left) and Sle1 Yaa (bottom left) mice. Ki67^+^Bcl6^+^ B cells are located at the T-B border in the T cell zone of male Sle1 TNF^–/–^ (top right) and Sle1 Yaa TNF^–/–^ (bottom right) mice (representative of 3–4 mice per group). (**J**) Ki67^+^ cells are located in GCs in Sle1 Yaa mice and at the T-B border and in the bridging zones adjacent to extrafollicular foci in Sle1 Yaa TNF^–/–^ mice. (**K**) Pie charts show mutation frequencies in VH sequences from CD138^+^ PCs from Sle1 Yaa (left) and Sle1 Yaa TNF^–/–^ (right) mice. χ^2^, ****P < 0.0001. (**L** and **M**) Percentage and count of CD19^+^CD11c^+^ age-associated B cells (ABC) in male Sle1, Sle1 Yaa, Sle1 TNF^–/–^, and Sle1 Yaa TNF^–/–^ mice. (**N** and **O**) Percentage and number of CCR6^+^CD38^+^ memory B cells in Sle1 Yaa and Sle1 Yaa TNF^–/–^ mice. Dots on bar graphs represent individual mice. Immunohistochemistry representative of 3–5 mice per group. (**B**–**H**, **L**, and **M**) ANOVA Kruskal-Wallis with Dunn’s multiple comparisons test, *P < 0.05: **P < 0.01, ***P < 0.001. (**N** and **O**) Mann-Whitney nonparametric test.

TLR7 is a ssRNA sensor whose overexpression in normal mice is sufficient to induce a lupus phenotype (49). We therefore asked whether excess TLR7 would induce GCs and pathogenic autoimmunity in Sle1 TNF^–/–^ mice by introducing the *Yaa* locus that codes for an extra copy of TLR7 on the Y chromosome. Male Sle1 Yaa mice have a vast expansion of GCs and high titers of somatically mutated autoantibodies against chromatin, Sm/RNP, and CL, and they develop fatal nephritis with age (50). Sle1 Yaa TNF^–/–^ mice manifested renal Ig and C3 deposition, proteinuria, and glomerular injury (Supplemental Figure 5, A–D). Kidneys from Sle1 Yaa TNF^–/–^ mice had fewer interstitial infiltrates than those from Sle1 Yaa mice (Supplemental Figure 5D), indicating a role for TNF in the effector phase of renal injury. However, mortality was similar in the 2 strains (Figure 6A).

Sle1 Yaa TNF^–/–^ mice developed earlier and higher titer anti-Sm/RNP and anti-cardiolipin autoantibodies than Sle1 Yaa mice (Figure 6, B–D). However, unlike control Sle1 Yaa mice, which had a significant increase in GC B cells, Sle1 TNF^–/–^ Yaa mice did not form GC structures and had only a modest increase in CD95^+^GL7^+^ cells that failed to upregulate PNA binding and Bcl6 or downregulate expression of CD38. In addition, fewer cells expressed the proliferation marker Ki67 (Figure 6, E–H; and Supplemental Figure 5, E and F). Immunohistochemical examination of Sle1 Yaa TNF^–/–^ spleens showed that Bcl6^+^Ki67^+^ B cells were located at the T-B border and in the bridging zones of the B cell follicles, adjacent to large extrafollicular clusters of PCs (Figure 6, I and J). Sequences of VH genes from single PCs from these mice showed the presence of abundant somatic mutations although at a lower frequency than in PCs from control Sle1 Yaa mice (Figure 6K). Sle1 Yaa TNF^–/–^ mice developed both age-associated B cells and CCR6^+^CD38^+^ memory B cells, similar to their Sle1 Yaa counterparts (Figure 6, L–O). Together, these data show that a strong innate stimulus can elicit pathogenic somatically mutated autoantibodies of extrafollicular origin, memory B cells, and fatal renal injury in the absence of both TNF and GCs.

### T-dependent immunization does not rescue the GC defect in TNF-deficient mice.

To determine whether activation of the adaptive immune system would induce pathogenic autoantibodies, we immunized 8- to 12-week-old mice with sheep red blood cells (SRBCs) either with or without a concomitant low dose of adenovirus expressing IFN-α that enhances GC formation (51). IFN-α markedly increased the GC response to SRBCs in Sle1 mice but did not increase the frequency of GCs in Sle1 TNF^–/–^ mice above baseline (Supplemental Figure 6) or cause premature mortality.

### Genetic background contributes to the effect of TNF deficiency on breach of B cell tolerance.

**Figure 7:**
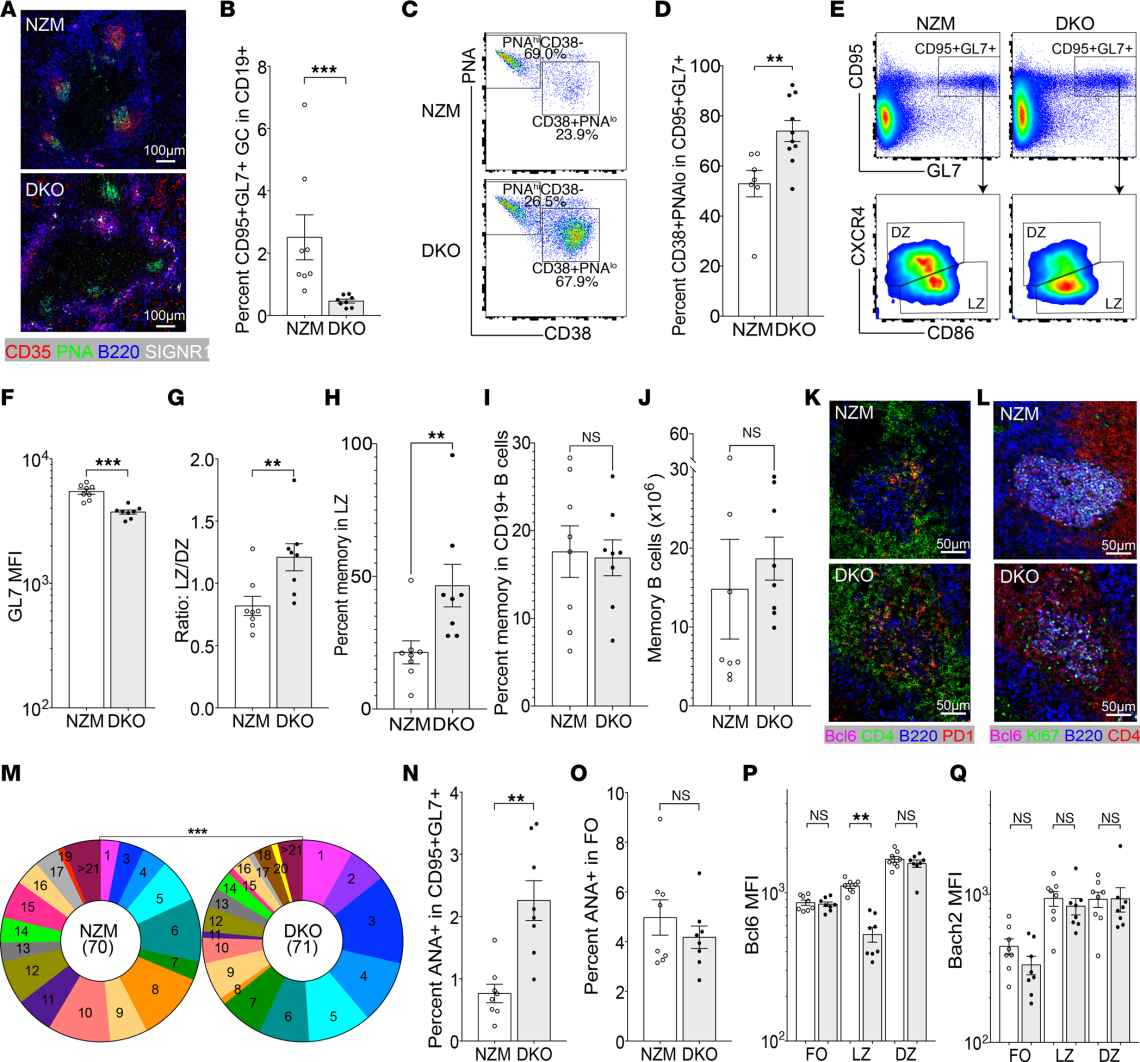
Atypical GCs in NZM2328 DKO mice. (**A**) PNA^+^ GC clusters (original magnification, 10×) in NZM2328 mice (top) and PNA^+^ clusters without FDCs in DKO mice (bottom). (**B**). Percentage CD95^+^GL7^+^ B cells in CD19^+^ B cells in nephritic NZM and DKO mice. (**C** and **D**) CD95^+^GL7^+^ B cells include PNA^hi^CD38^lo^ GC B cells and PNA^lo^CD38^+^ memory cells. (**E**) Flow plots show the gating of splenic CD95^+^GL7^+^ B cells in CD19^+^ B cells from NZM and DKO mice and their LZ and DZ phenotype. (**F** and **G**) Bar graphs show the summary result of GL7 expression (**F**) and LZ/DZ ratio (**G**). (**H**) Percentage CCR6^+^CD38^+^ memory B cells in LZ CD95^+^GL7^+^CXCR4^–^CD86^+^ compartment in NZM and DKO mice. (**I** and **J**) Percentage (**I**) and number (**J**) of CCR6^+^CD38^+^ memory B cells in NZM and DKO mice. (**K**) Immunohistochemistry images (original magnification, 20×) show PD1^hi^CD4^+^ T cells in DZ of GCs of NZM (top) and scattered throughout the GC cluster in DKO (bottom) mice. (**L**) Immunohistochemistry images (original magnification, 20×) show Ki67^+^Bcl6^+^ B cells in GCs of NZM (top) and DKO (bottom) mice (representative of 3–4 mice per group). (**M**) Pie charts show mutation frequencies in VH sequences from CD95^+^GL7^+^ B cells from NZM (left) and DKO (right) mice. χ^2^, ***P < 0.001. (**N** and **O**) Percentage ANA-positive B cells in CD95^+^GL7^+^ GC B cells (**N**) and follicular IgM^+^IgD^+^ B cells (**O**). (**P** and **Q**) Bar graphs show the summary result of Bcl6 (**P**) and Bach2 (**Q**) expression on CD95^+^GL7^+^ GC B cells. NZM mice (white bars); DKO mice (gray bars). Dots on bar graphs represent individual mice. Immunohistochemistry representative of 3–5 mice per group. (**B**, **D**, **F**–**J**, **N**, and **O**) Mann-Whitney nonparametric *t* test, **P < 0.01, ***P < 0.001. (**P** and **Q**) ANOVA Kruskal-Wallis with Dunn’s multiple comparisons test, **P < 0.01.

We next addressed whether a more complex strong genetic predisposition to SLE would enhance the development of GCs and/or pathogenic antibodies in the absence of TNF. It has previously been reported that lupus-prone NZM mice deficient in both TNFR1 and TNFR2 (double knockout, DKO) develop accelerated lupus with anti-dsDNA antibodies and expansion of TH17 cells and of PNA^+^ GC-like structures (39). Although we observed clusters of PNA^+^ cells within the B cell follicles in DKO mice as previously reported (39), they did not contain FDCs (Figure 7A), and GL7 could not be detected using immunohistochemistry. Flow cytometric analysis of spleens from DKO mice revealed CD95^+^GL7^+^ B cells (Figure 7B), but, as in curli-treated Sle1 TNF^–/–^ mice, many of these cells were PNA^lo^CD38^+^ (Figure 7, C and D), the median fluorescence intensity for GL7 was low, and the cells were skewed toward an LZ phenotype (Figure 7, E–G), with an increase in frequency of CCR6^+^CD38^+^ cells within the LZ compartment (CD95^+^GL7^+^CXCR4^–^CD86^+^ — Figure 7H). The percentage and number of total memory B cells (CD19^+^CCR6^+^CD38^+^) were preserved in DKO spleens compared with NZM controls (Figure 7, I and J). CD4^+^ T cells were diffusely distributed within the clusters of PNA^+^ B cells (Figure 7K), and the B cells within the clusters expressed both Ki67 and Bcl6 (Figure 7L). A high frequency of somatic mutations was found in the heavy chains of CD95^+^GL7^+^ B cells sorted from DKO mice albeit at a lower frequency than in NZM wild-type mice (Figure 7M). Importantly, there was a higher frequency of ANA^+^ B cells among the atypical GC B cells of DKO mice compared with NZM mice but no difference in ANA reactivity in the follicular B cell subset (Figure 7, N and O). Thus, despite the absence of FDCs or a defined DZ or LZ, the atypical clusters of PNA^+^ cells found in DKO mice can undergo somatic mutation and have an additional defect in negative regulation of autoreactive B cells.

Both Bcl6 and BTB domain and CNC homolog 2 (Bach2) are needed to sustain the GC program. Bcl6 is expressed in the DZ but downregulated in the LZ because of repression by CD40L signaling, whereas Bach2 expression is inversely proportional to the amount of T cell help (52–54). Bcl6 downregulation appeared more robust in the LZ cells of DKO mice, perhaps reflecting the excess T cell activation in these mice (Figure 7P). Nevertheless, we found no difference in Bach2 expression between GC cells from NZM and DKO mice (Figure 7Q).

### Failure of activated B cells to acquire high GL7 expression is due to altered remodeling of glycosylation in TNF^–/–^ mice and is not B cell intrinsic.

The acquisition of both PNA and GL7 positivity of GC B cells is due to remodeling of glycosylation of cell surface proteins, including CD45 and CD22. In mice, the acquisition of GL7 positivity is mediated by a single enzyme, CMP-Neu5Ac hydroxylase (CMAH), that catalyzes the conversion of Neu5Ac to Neu5Gc, generating high-affinity ligands for CD22 to bind in *cis* (55, 56). GL7 is a rat monoclonal antibody that recognizes Neu5Ac glycans (55). We confirmed, using both flow cytometry and immunohistochemistry, that downregulation of Neu5Gc expression was significantly less in the CD95^+^GL7^lo^ B cells of Sle1 TNF^–/–^ mice or within atypical CD95^+^GL7^+^ GC cells of DKO mice than in the CD95^+^GL7^+^ GCs of C57BL/6, Sle1, and NZM mice (Supplemental Figure 7, A–E). In vitro culture of splenic follicular B cells from Sle1 TNF^–/–^ mice with feeder cells expressing CD40L, IL-21, and BAFF (57) showed comparable upregulation of GL7 expression as in Sle1 mice (Supplemental Figure 7, F and G), showing that TNF^–/–^ B cells are not intrinsically defective in CMAH function.

## Discussion

Although the frequent induction of autoantibodies by TNF inhibitors has long been recognized, the mechanism for this side effect is not known, nor is it understood why only a limited subset of patients develop clinical disease. Even more puzzling has been the development of autoantibodies and lupus in patients treated with TNF inhibitors despite the robust inhibitory effect of these drugs on the GC, a site where pathogenic autoantibodies are often generated. Our studies in informative mouse models shed light on the mechanisms both for autoantibody production and for progression to clinical disease in patients treated with TNF inhibitors.

The Sle1 mouse contains a genetic locus that confers loss of tolerance to chromatin and other lupus-related antigens without overt clinical disease. We show here that TNF deficiency is not sufficient to induce either GC formation or pathogenic autoimmunity in Sle1 mice. TNF deficiency in Sle1 mice resulted in a decrease in the production of anti-chromatin autoantibodies that are of GC origin while breaching tolerance to CL in the extrafollicular compartment.

**Figure 8:**
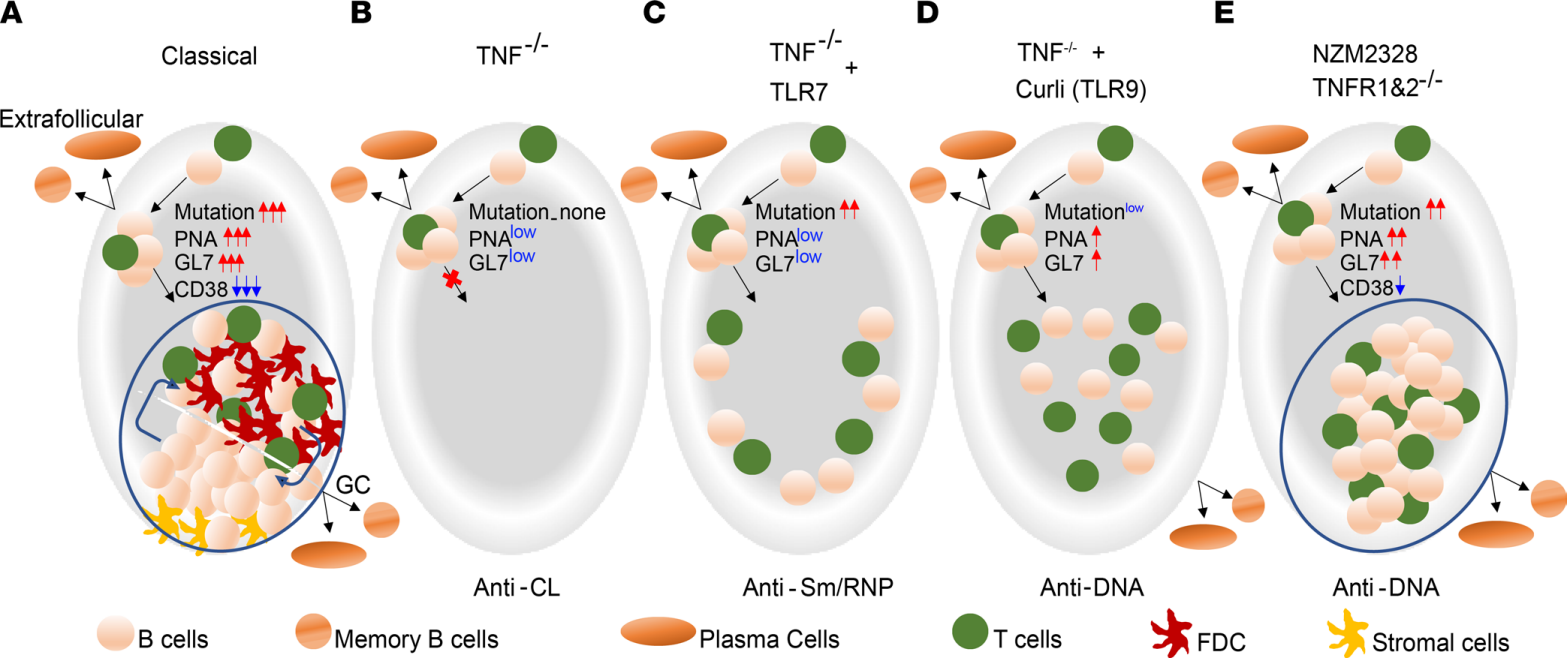
Model of diverse mechanisms for induction of autoreactivity by TNF deficiency. (**A**) Typical GC in Sle1 mice. (**B**) TNF deficiency alone induces germline encoded autoantibodies from extrafollicular sites. (**C**) With TLR7 overexpression, a further breach of B cell tolerance occurs extrafollicularly; these autoreactive B cells are highly mutated and pathogenic. (**D**) TLR9 stimulus induces atypical, activated B cells that stay scattered in the T cell zone and have a low frequency of somatic mutations. (**E**) TNF deficiency in the NZM2328 model induces atypical GC clusters that are missing crucial negative signals that help to regulate autoreactivity. Activated autoreactive B cells in these atypical structures are highly mutated and are associated with accelerated disease onset.

Autoantibodies that cause SLE flares can arise from either an extrafollicular pathway or a GC pathway (58, 59). We found that TNF deficiency impaired B cell tolerance by several mechanisms depending on the strain of the mouse and the nature of the extrinsic stimulus (Figure 8). In Sle1 mice, both pristane administration and introduction of an extra copy of TLR7 (*Yaa* locus) induced anti-Sm/RNP antibodies via the extrafollicular pathway, but only those found in mice with the *Yaa* locus were associated with a high frequency of somatic mutations and clinical disease. The absence of GCs did not impair memory B cell formation in these mice, showing that the extrafollicular memory B cell pathway (60) is intact in the absence of TNF signaling. By contrast, short-term administration of the TLR2/TLR9 agonist curli, a DNA-binding protein found in urinary tract pathogens, breached tolerance to DNA and chromatin. Activated CD95^+^GL7^+^ B cells induced by curli were neither classic extrafollicular cells nor GC cells but were scattered in the T cell zone and expressed a phenotype similar to that of activated B cells that enter the GC-independent early memory pathway (60, 61). Despite their proximity to T cells, class switching and somatic mutation in these B cells were limited. Repertoire analysis from curli-treated 3H9 transgenic mice suggested that the TLR2/9-mediated additional signal did not alter repertoire selection but enhanced B cell activation and expansion without reestablishing the GC network. These findings are similar to those recently reported in both lupus-prone mice and patients with SLE in which an increase in autoreactive B cells during disease flare is associated with general PC expansion (59). A different result was observed in NZM mice deficient in signaling through both TNFR1 and TNFR2. Here, accelerated development of anti-DNA autoantibodies and premature mortality were associated with the formation of atypical PNA^+^ clusters in which class switching and a high frequency of somatic mutation could occur.

Normal organization of GCs requires the presence of TNFR1 on FDCs and TNF on B cells (30, 31). This interaction induces the production of CXCL13 by FDCs that in turn stimulates lymphotoxin αβ production by B cells and the consequent expression of adhesion molecules that allow segregation of the GC into the LZ in which B cells interact with FDCs and receive T cell help and the DZ in which they undergo proliferation and extensive somatic mutation. Segregation into DZ and LZ is associated with the upregulation of CXCR4 on DZ B cells and their interaction with CXCL12-expressing DZ stromal cells. This organization enhances selection of high-affinity B cells and also helps maintain B cell tolerance by delivering negative signals via both FDCs and T cells that prevent the emergence of autoimmunity as a result of overexpansion of the GC and/or excess somatic mutations (35, 62). The atypical PNA^+^ GC-like clusters in DKO mice lacked FDCs and contained B cells that were skewed toward an LZ (CXCR4^lo^) phenotype and are reminiscent of those found in CXCL12-deficient mice (53). The GCs that form after immunization of CXCL12-deficient mice with the T-dependent hapten NP-CGG exhibit fewer somatic mutations and less clonal diversity after immunization, suggesting either that effective GC responses are attenuated because of the absence of a DZ or that the B cells are diverted to the GC-derived memory pathway because of inadequate access to antigen or T cell help (61, 63). Our finding of a skewing to the CCR6^+^CD38^+^ memory phenotype within the LZ of DKO mice is consistent with the latter view. Similar findings have been reported in mice in which FDCs have been rendered deficient in the negative signaling molecule Fcγ receptor IIB (FcγRIIB) (64). Importantly, although atypical GC B cells in DKO mice similarly accumulated fewer somatic mutations than those from the control NZM mice, these cells manifested an increase in the percentage of ANA-binding B cells. This defect in negative selection could be responsible for the pathogenic autoimmunity and early mortality observed in DKO mice.

A shared characteristic of activated B cells in both curli-treated Sle1 TNF^–/–^ and DKO mice was their failure to downregulate Neu5Gc, a defining feature of glycosylation remodeling of GC B cells that is accompanied in mice by the acquisition of high levels of the cell surface marker GL7. Conversion of Neu5Ac to Neu5Gc by the enzyme CMAH generates high-affinity ligands for the negative regulatory molecule CD22 (sialic acid-recognizing Ig superfamily member Siglec-2) to bind in *cis*. Upon B cell activation, CMAH is downregulated, there is a decrease in Neu5Gc, and CD22 is unmasked, allowing it to bind in *trans* to ligands on other cells, such as CD4^+^ T cells. This receptor unmasking allows CD22 to move into close proximity to the B cell receptor (BCR) complex where Lyn tyrosine kinase phosphorylates CD22’s immunoreceptor tyrosine-based inhibition motif, to recruit Src homology region 2 domain–containing phosphatase-1 and negatively regulate BCR signals (55, 56, 65, 66), thereby modulating the threshold for B cell activation. Coengagement of CD22 and the BCR suppresses BCR signaling and can induce antigen-specific B cell tolerance and induction of B cell death via induction of BIM (67, 68). While the exact mechanisms by which remodeling of CD22 ligands in GC B cells influence their function are still not fully understood (65), the reduction of the CD22-mediated negative signal in atypical GC cells, together with a functional defect in the negative signaling molecule FcγRIIB due to absence of FDCs, could contribute to the attenuation of negative selection in TNF signaling–deficient GC clusters. Notably, we show that the CMAH defect is not intrinsic to TNF-deficient B cells since they can acquire GL7 normally when exposed to an appropriate microenvironment. Although CMAH is not present in humans, glycosylation of CD22 and its ligands is similarly mediated by enzymes whose expression is regulated by TNF (69–71).

Another possible reason for loss of tolerance in TNF-deficient mice is the failure to regulate activated T cells. Because AICD of effector T cells is in part TNF dependent, TNF deficiency results in expansion of this population, including TH17 cells (39). The failure to regulate autoreactivity in the absence of TNF may also reflect the disorganization of the follicle so that TFH and TFR cells are not situated within an organized microenvironment that optimally fosters normal T cell regulatory mechanisms.

Our data, in sum, show that TNF deficiency can be associated with loss of B cell tolerance in multiple sites, including the extrafollicular region, inside follicles, and in atypical GCs that fail to segregate into DZ and LZ regions and to remodel B cell glycosylation. The outcome of TNF deficiency in Sle1 mice is the induction of nonpathogenic autoreactivity despite T cell activation, but, as shown here, both a permissive genetic background and the introduction of extrinsic innate signals induce atypical, activated B cells that accumulate somatic mutations and are associated with pathogenic autoreactivity (Figure 8). Notably, TNF deficiency is not sufficient to prevent tissue damage and mortality due to nephritis in genetically predisposed models.

Our findings help explain the variability of the autoreactive B cell response in patients exposed to TNF inhibitors. Our data suggest that class switching and somatic mutation of autoreactive B cells will be hallmarks of risk for clinically relevant autoimmune side effects and that aberrant remodeling of B cell glycosylation could contribute to altered B cell selection. We are currently examining these pathways in patients who are initiating anti-TNF treatment.

## Methods

### Mice.

C57BL/6 CD45.1 congenic mice, C57BL/6 Yaa mice; and mice deficient in TNF, TNFR1, and TNFR2 were obtained from The Jackson Laboratory and backcrossed to Sle1 and Sle1 Yaa mice to generate Sle1, Sle1 Yaa, Sle1 Yaa CD45.1, Sle1 TNF^–/–^, Sle1 Yaa TNF^–/–^, Sle1 TNFR1^–/–^, Sle1 TNFR2^–/–^, and Sle1 TNFR1&2^–/–^ strains. NZM and NZM2328 TNFR1/TNFR2 DKO mice were a gift from Chaim Jacob, University of Southern California, Los Angeles, California, USA. Male and female mice of the various strains were aged to more than 1 year and were followed at regular intervals for proteinuria and autoantibody production. To determine the effect of TNF and TNFR deficiency on selection of the autoreactive B cell repertoire, C57BL/6 mice bearing the autoreactive 3H9 heavy chain transgene (a gift of Robert Eisenberg, University of Pennsylvania, Philadelphia, Pennsylvania, USA; and Martin Weigert, University of Chicago, Chicago, Illinois, USA) were backcrossed to the Sle1 and Sle1 TNF^–/–^ strains (3H9 Sle1 and 3H9 Sle1 TNF^–/–^).

### Quantitation of autoantibodies against chromatin, CL, Sm/RNP, and dsDNA.

Serial ELISAs of serum for antibodies against CL/β2GP1, Sm/RNP and dsDNA were performed as previously described (72–74) using test sera dilutions of 1/250. For anti-CL/β2GP1 ELISAs using 3H9 mice, test sera were diluted to 1/500. Antibodies against chromatin were measured by ELISA as described by Mohan et al. (75) using test sera dilutions of 1/500. A high-titer serum was run in serial dilutions on each plate. Using this control as a standard, OD values were converted to units using regression analysis (GraphPad Prism Version 9), and data were expressed as arbitrary units compared with this control. Maximal units set for each ELISA correspond with the test serum dilution and represent the OD at which the plateau for the ELISA is reached.

### Flow cytometry.

Mice of both sexes were euthanized, and spleen cell subsets were analyzed as previously described (51, 76, 77). After exclusion of doublets and dead cells, CD19^+^ B cells subsets were identified as transitional T1 (CD21^hi^CD23^–^IgM^hi^IgD^lo^), transitional T2 (CD21^+^CD23^+^IgM^hi^IgD^lo^), follicular (FO, CD21^+^CD23^+^IgM^+^IgD^+^), marginal zone (MZ, CD21^hi^CD23^–^IgM^+^IgD^+^), and GC (CD95^+^GL7^+^). GC B cells were further divided into DZ (CXCR4^hi^CD86^lo^) and LZ (CXCR4^lo^CD86^hi^) phenotypes. PCs were identified as CD138^+^IgD^–^. Memory cells were defined as CCR6^+^CD38^+^ (78). Subsets of CD3^+^CD4^+^ T cells were identified as activated (CD4^+^CD44^+^PD1^hi^PSGL1^–^), TFH (CD4^+^CD44^+^PD1^hi^PSGL1^–^Bcl6^+^FoxP3^–^), and TFR (CD4^+^CD44^+^PD1^hi^PSGL1^–^Bcl6^+^FoxP3^+^) phenotypes. Other T cell subsets were examined as indicated in Table 1. For IL-17A staining, spleen cells were stimulated with or without PMA/ionomycin (1 μg/mL) in the presence of GolgiStop Protein Transport Inhibitor (BD Biosciences) for 4 hours before being loaded with Live/Dead dye (BioLegend), fixed, permeabilized, and stained. Antibodies and their sources are listed in Supplemental Table 1.

### Treatment with anti-CD4 and TACI-Ig.

Mice that were 5–6 months old were treated for 8 weeks with anti-CD4 1 mg weekly or TACI-Ig 200 μg 3 times per week (77). Autoantibody titers were measured after 21, 27, and 48 days. After 8 weeks, mice were euthanized and ELISPOT analyses were performed on spleen cells as previously described to enumerate Ig-secreting and anti-cardiolipin–secreting B cells (74).

### Immunizations and innate immune challenge.

Mice were immunized with 10^9^ SRBCs (Cocalico Biologicals) in PBS with or without a single low dose of adenovirus expressing IFN-α (3 × 10^8^ particles) given 72 hours prior to SRBC immunization (51). To mimic an innate immune challenge, Sle1 and Sle1 TNF^–/–^ mice were given a single intraperitoneal dose of pristane (500 μL) and were followed for up to 6 months. Alternatively, Sle1 and Sle1 TNF^–/–^ mice were treated intraperitoneally with the TLR2/TLR9 agonist curli protein 50 μg twice weekly for 2 weeks (47).

### Renal pathology and immunohistochemistry.

H&E sections of kidneys from >9-month-old Sle1 Yaa and Sle1 Yaa TNF^–/–^ mice were prepared and displayed as digital images by HistoWiz. Glomerular damage and interstitial infiltrates were scored on a semiquantitative scale from 0 to 6 while blinded to the mouse genotype. Eight-micrometer kidney sections were stained with anti-IgG PE (catalog 31861, Invitrogen) and anti-C3 FITC (clone RmC11H9, Cedarlane).

Eight-micrometer spleen sections were stained with 4-color panels using combinations of anti-B220, anti-IgD, anti-CD138, anti–SIGN-R, anti-Neu5Ac (GL7), anti-CD21/35, anti-CD4, anti-Ki67, anti-Bcl6, anti-FoxP3, anti–PD-1, anti-Neu5Gc, and PNA (Supplemental Table 1). Images were obtained using a Zeiss Apotome confocal microscope.

### Single-cell sorting and PCR.

Single MZ, FO, and/or GC B cells and/or PCs were sorted as previously described (43, 79) from 4 to 6 3H9 transgenic mice of Sle1, Sle1 TNF^–/–^, and Sle1 TNFR2^–/–^ strains; from 3 curli-treated Sle1 and 3 Sle1 TNF^–/–^ mice; and from 3 mice each of Sle1 Yaa, Sle1 Yaa TNF^–/–^, NZM, and DKO strains. cDNA preparation and PCR of the 3H9 heavy chain and associated Vk light chain were performed as previously described, using a constant region IgM primer for naive and MZ cells and a constant region IgG primer for GC cells and PCs (43, 79). PCR products were sequenced by GENEWIZ, and sequences were identified using the International ImMunoGeneTics database. Full-length heavy chains from cells encoded by Vk57*1*01 were reamplified using a Vk57*1*01 leader region 5*′* primer.

### Statistics.

Survival data in Figures 1 and 6 and Supplemental Figure 5 were analyzed using Kaplan-Meier curves and log-rank test. Comparisons of more than 2 groups in the bar graphs shown in Figures 1–7 and Supplemental Figures 1 and 4–7 were performed using the Kruskal-Wallis nonparametric 1-way ANOVA. Upon obtaining a significant result of *P* < 0.05, pairwise multiple comparison was carried out using Dunn’s multiple comparisons test based on the number of predetermined comparisons. Data in Figure 2H were analyzed using ordinary 2-way ANOVA with Tukey’s multiple comparisons test. Analysis of the Vk repertoire data shown in Figure 3 and Supplemental Figure 4 was performed using Fisher’s test of rxc (rows × columns) tables using R package (Fisher.test{stats} with Monte Carlo simulation, 2000 replicas). Overrepresented Vk genes were identified using the statistical program previously described in detail (45). Comparisons of somatic mutation frequencies were performed using χ^2^ analysis. Statistical methods for each figure and significant *P* values are indicated in the figure legends.

### Study approval.

All studies were approved by the Northwell Health IACUC.

## Author contributions

AD and TDQ designed and performed experiments, analyzed data, and wrote the manuscript; WH, RS, CMMD, and PGR performed experiments; CR performed and analyzed results of single-cell experiments; RJ performed and analyzed T cell–dependent experiments; SM provided expertise on ANA reagent; SG and ÇT provided curli reagent and read the manuscript; COJ provided NZM and DKO mice and read the manuscript; YZ consulted on experimental design and edited the manuscript; and TL and MLL performed the statistical analyses.

## Supplementary Material

Supplemental data

## Figures and Tables

**Table 1:**
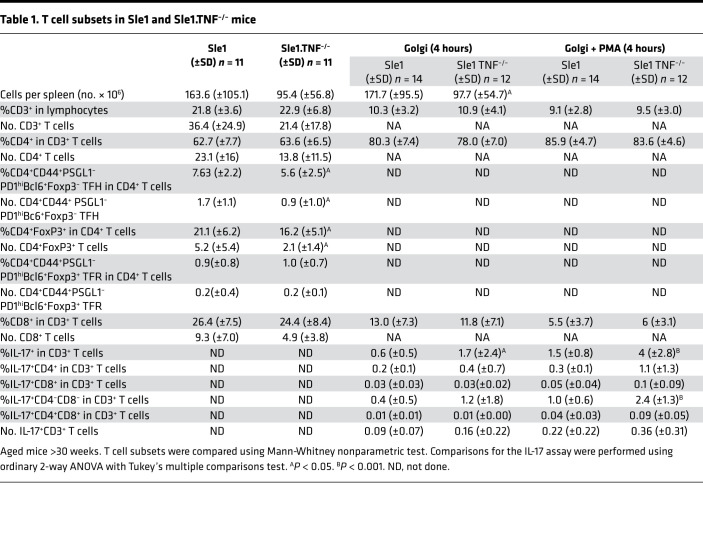
T cell subsets in Sle1 and Sle1.TNF^–/–^ mice

## Version 1 02/01/2022

In-Press Preview

## Version 2 03/08/2022

Electronic publication
