# Supplementary material for: Context-dependent induction of autoimmunity by TNF signaling deficiency
Source: JCI Insight. 2022 Mar 8;7(5):e149094. doi: 10.1172/jci.insight.149094 (PMC8983147; doi:10.1172/jci.insight.149094)
Supplement: Supplemental data [file jciinsight-7-149094-s092.pdf]

Context dependent induction of autoimmunity by TNF signaling deficiency

SUPPLEMENTARY TABLES, FIGURES, AND LEGENDS

Supplementary Table 1: Antibodies and reagents

Reagent	Fluorochromes	Clone	Vendor
CD3	PE Dazle 594/AF700	17A2	Biolegend
CD4	BV650	RM4-5	Biolegend
CD8	BV711	53-6.7	Biolegend
CD19	APC/F750	6D5	Biolegend
CD45R/B220	FITC/BV421/BV785	RA3-6B2	Biolegend
CD21/CD35	BV605/Biotin/AF647	7G6	BD
CD23	BV711/PE-Cy7/BUV736	B3B4	BD
CD38	AF700/BUV395	90/CD38	BD
CD44	BV785	IM7	Biolegend
CD45.1	AF488/PE/APC	A20	eBiosciences/Southern
CD45.2	AF700/PerCP-Cy5.5	104	Biolegend
CD62L	BV605	MEL-14	Biolegend
CD86	BV785	GL-1	Biolegend
CD95	PE-Cy7/AF700	Jo2	BD
CD138	BV421/PE/BV711	281-2	Biolegend
ANA	Biotin	NA	Lab made
BACH2	unconjugated	Polyclonal	Novus
Goat anti-Rabbit	PE	Polyclonal	Southern Biotech
Bcl6	AF647	K112-91	BD
CXCR4	PerCP-Cy5.5	L276F12	Biolegend
CXCR5	Biotin		
FoxP3	AF488	R16-715	BD
GL7	FITC/AF647/BV421	GL7	BD
Ki67	FITC/AF647/AF700/BV421	16A8	Biolegend
IL17A	PerCP-Cy5.5		
IgD	FITC/PE/BV650	11-26c.2a	Biolegend
IgM	PE/PE-D594/BV650		BD/Biolegend
LiveDead Dye	Aqua fluorescent	NA	Invitrogen/ThermoFisher
Neu5Gc	unconjugated	Poly21469	Biolegend
Goat anti-chicken IgY	FITC	Poly24108	Biolegend
PD1	PE-Cy7	RMP1-30	Biolegend
PNA	Fluorescein/Cy5		Vector Laboratories
PSGL1	BV421	2PH1	BD
Streptavidin	PE/PerCP-Cy5.5/FITC/PB/PE-Cy7	NA	BD
CD11b	AF700/PB	M1/70	Biolegend
CD11c	AF700	HL3	BD
CD25	PerCP-Cy5.5	3C7	Biolegend
CCR6	BV650	11A9	BD

6

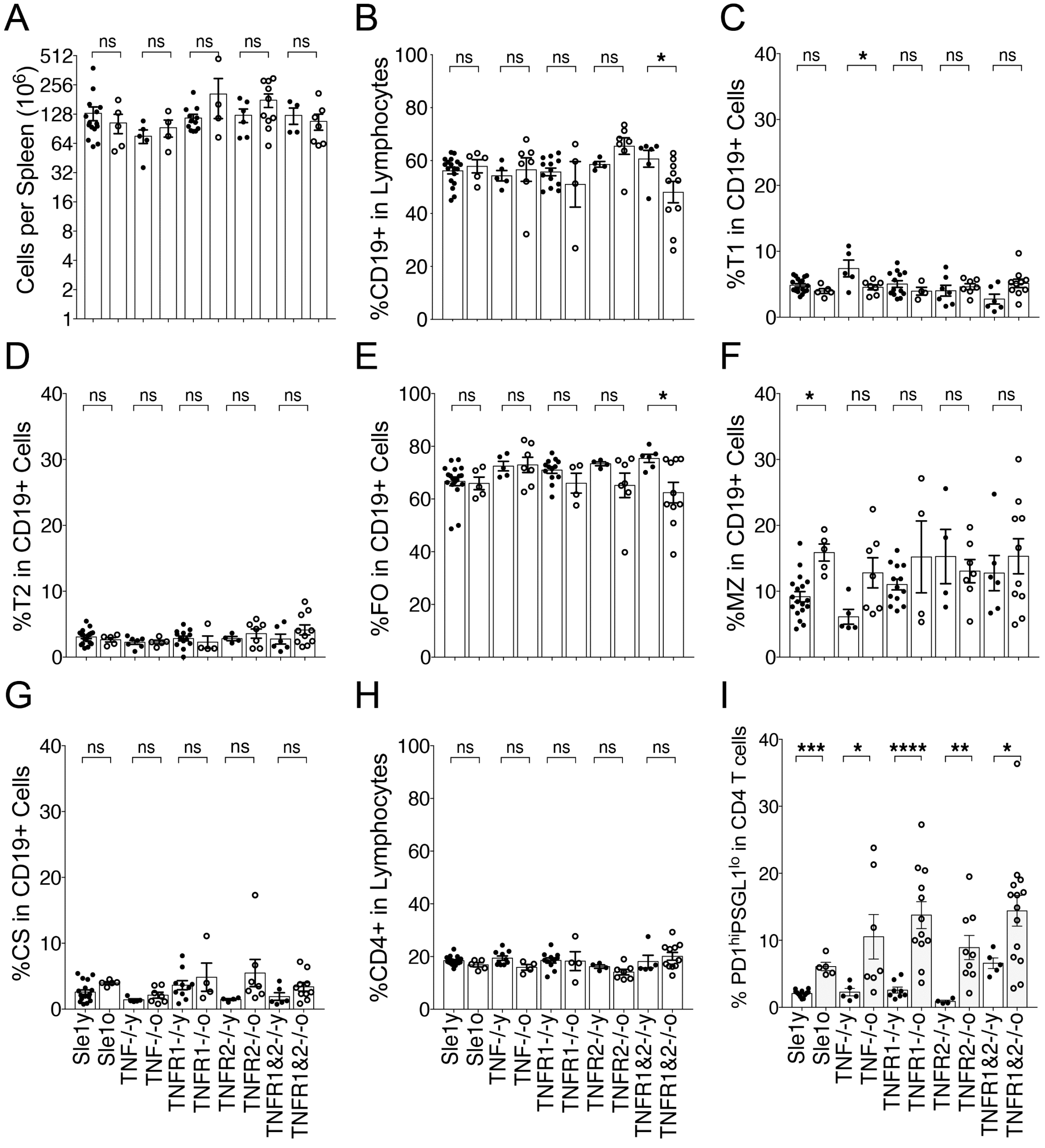

A
B
C
D
E
F
G
H
I
ns
*
**
***
****
Cells per Spleen (10^6)
%CD19+ in Lymphocytes
%T1 in CD19+ Cells
%T2 in CD19+ Cells
%FO in CD19+ Cells
%MZ in CD19+ Cells
%CS in CD19+ Cells
%CD4+ in Lymphocytes
% PD1hiPSGL1lo in CD4 T cells
Sle1y
Sle1o
TNF-/-y
TNF-/-o
TNFR1-/-y
TNFR1-/-o
TNFR2-/-y
TNFR2-/-o
TNFR1&2-/-y
TNFR1&2-/-o

7

Supplementary Figure 1. TNF deficiency does not alter B cell phenotype or prevent T cell activation in Sle1 mice. **A-I.** Bar graphs show the number of spleen cells (A), percent of $CD19^+$ (B), transitional T1 (C), T2 (D), Follicular (FO, E), Marginal Zone (MZ, F), IgM^-IgD^- class switched (CS, G) B cells, $CD4^+$ T cells (H), and $CD4^+CD44^+PSGL1^-PD1^{hi}$ activated T cells (I) from Sle1 mice of the indicated genotypes. Dots represent individual mice. No differences between the two strains were observed. Mann-Whitney t-test comparing young and aged mice of each strain. ANOVA Kruskal-Wallis with Dunn's multiple comparisons test, * $p<0.05$: ** $p<0.01$, *** $p<0.001$, *** $p<0.0001$, ns = not significant. y = young (2-3 months), o = old (> 6 months).

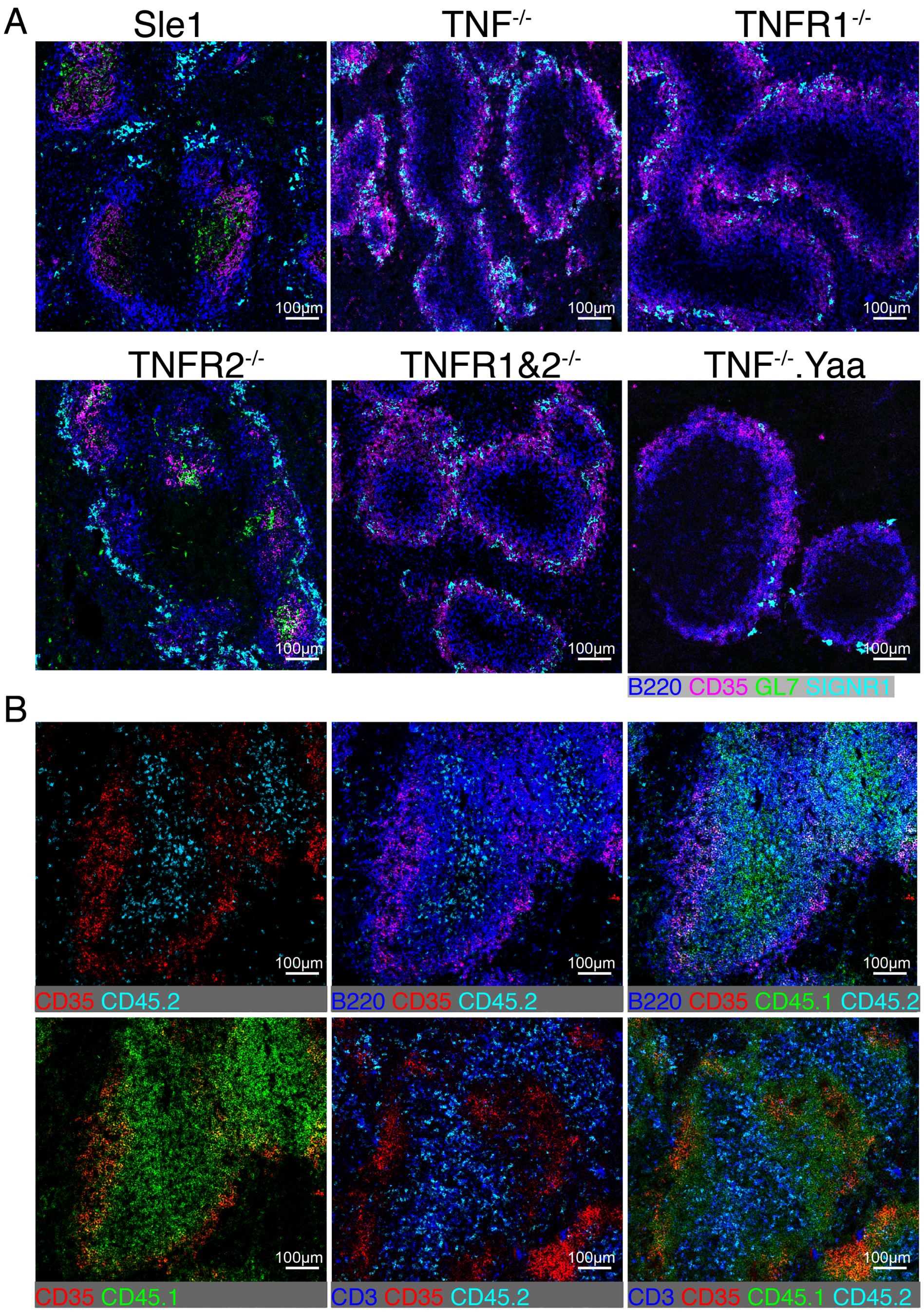

Supplementary Figure 2. CD21/35^{hi} cells in Sle1.TNF$^{-/-}$ and Sle1.TNFR1$^{-/-}$ mice are B cells. A. Immunohistochemistry images (10x) of Sle1 mice of the indicated genotypes show germinal center B cells (GL7), FDCs (CD35/21) and marginal zone macrophages (SIGNR1). GC clusters and FDCs are seen only in Sle1 and Sle1.TNFR2$^{-/-}$ mice. Note the location of CD35 positive cells in a ring around the follicle in TNF and TNFR1 deficient

mice. **B.** 3 x 10^6 bone marrow cells from Sle1 mice (CD45.1) were transplanted intravenously into Sle1.TNFR1-/- mice (CD45.2) 24 hours after lethal irradiation (950 rads) and recipients were euthanized after 3 months. Immunohistochemistry images of chimeric spleens show that CD35/21^{hi} cells in the ring around the follicle (red) are B cells of donor (CD45.1) origin (see co-expression of CD35 with B220 and co-expression of CD35 with CD45.1).

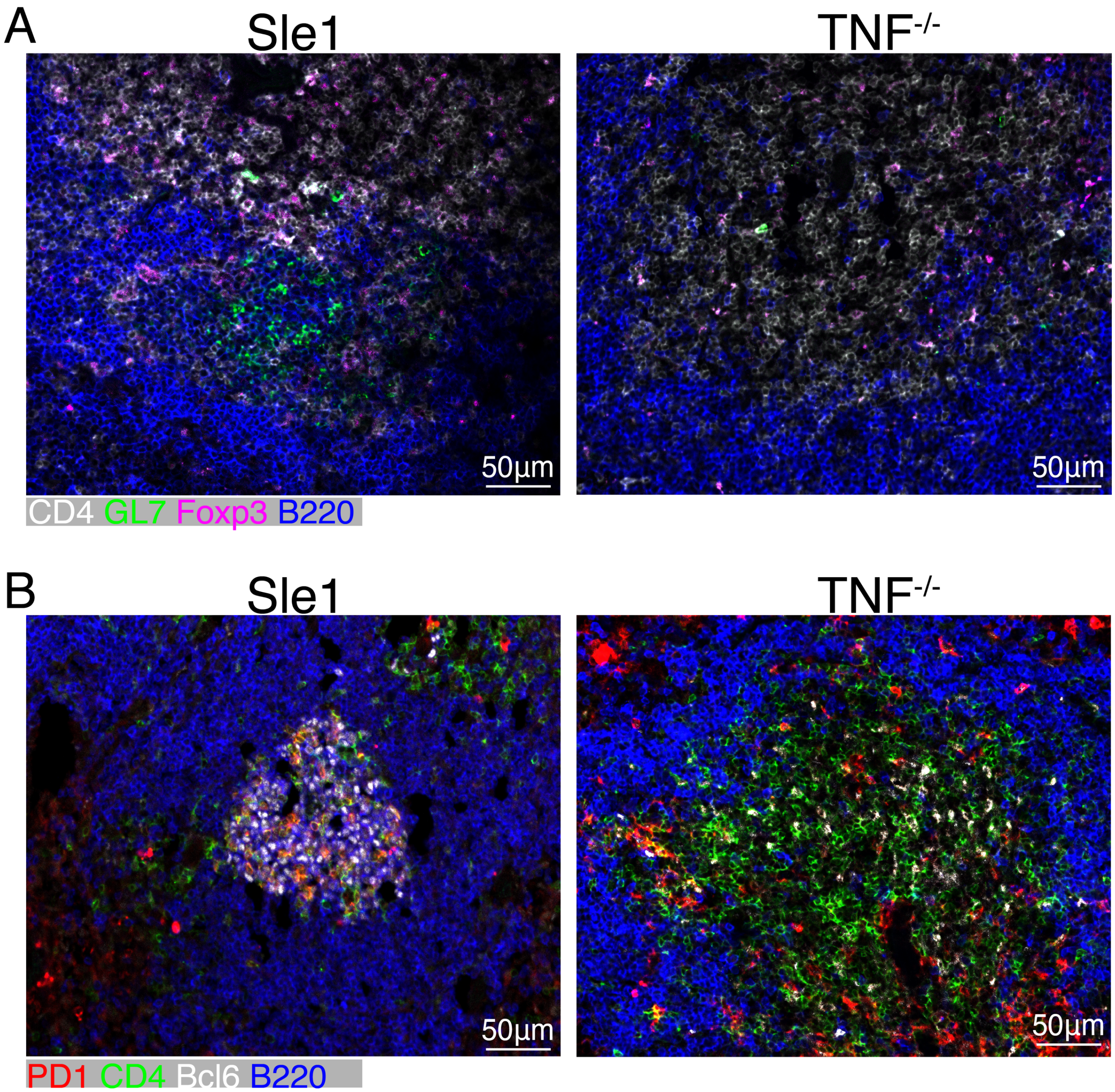

Supplementary Figure 3. Abnormal localization of CD4 T cells in Sle1.TNF$^{-/-}$ mice. A. Immunohistochemistry images (20x) show Foxp3^{+} T cells in the LZ of GCs in Sle1 mice but scattered localization in Sle1.TNF$^{-/-}$ mice. **B.** PD1^{+} and Bcl6^{+} positive CD4 T cells are located within the GCs of Sle1 mice but are scattered in the T cell zone of Sle1.TNF$^{-/-}$ mice.

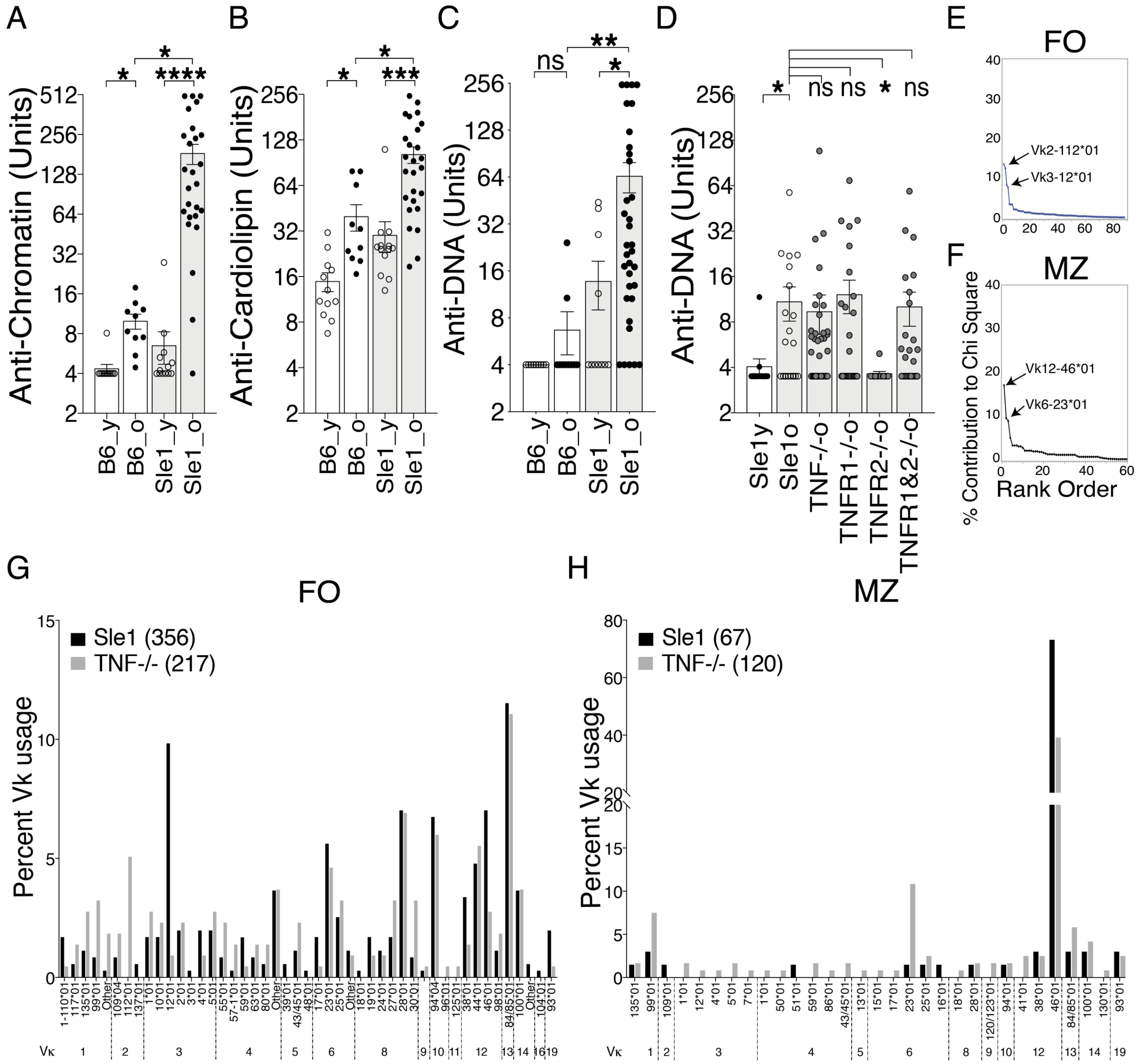

Supplementary Figure 4. Vk repertoire in B cell subsets of 3H9.Sle1.TNF$^{-/-}$ and 3H9.Sle1.TNFR2$^{-/-}$ mice. A-B. B6 control autoantibody profile and Vk repertoire in B cell subsets of 3H9.Sle1 and 3H9.Sle1.TNF$^{-/-}$ **A-C.** Bar graphs show the relative units of IgG antibodies to chromatin (B), CL/β2GP1 (C) and DNA (D) from sera of 2-3 months old (y) and > 6 months old (o) C57BL/6 and Sle1 mice. ANOVA Kruskal-Wallis with Dunn's multiple comparisons test, * p<0.05: ** p<0.01, *** p<0.001, *** p<0.0001, , ns = not

significant. y = young (2-3 months), o = old (> 6 months). **D.** Bar graphs show the relative units of IgG antibodies to dsDNA from sera of Sle1 mice of the indicated genotypes (y = 2-3 months old, o > 9 months old). **E-F**. Scree plots show the percent contribution to the chi-square analysis of the most overrepresented V*k* genes in $3H9^{+}$ follicular (E) and marginal zone (F) cells from 3H9.Sle1 versus $3H9.Sle1.TNF^{-/-}$ mice. **G-H.** Percent of Vk gene usage in $3H9^{+}$ follicular (G) and marginal zone (H) cells from 3H9.Sle1 and $3H9.Sle1.TNF^{-/-}$ mice. Fisher Exact test rxc table, $p = 0.671$ (E), $p<0.0004$ (F).

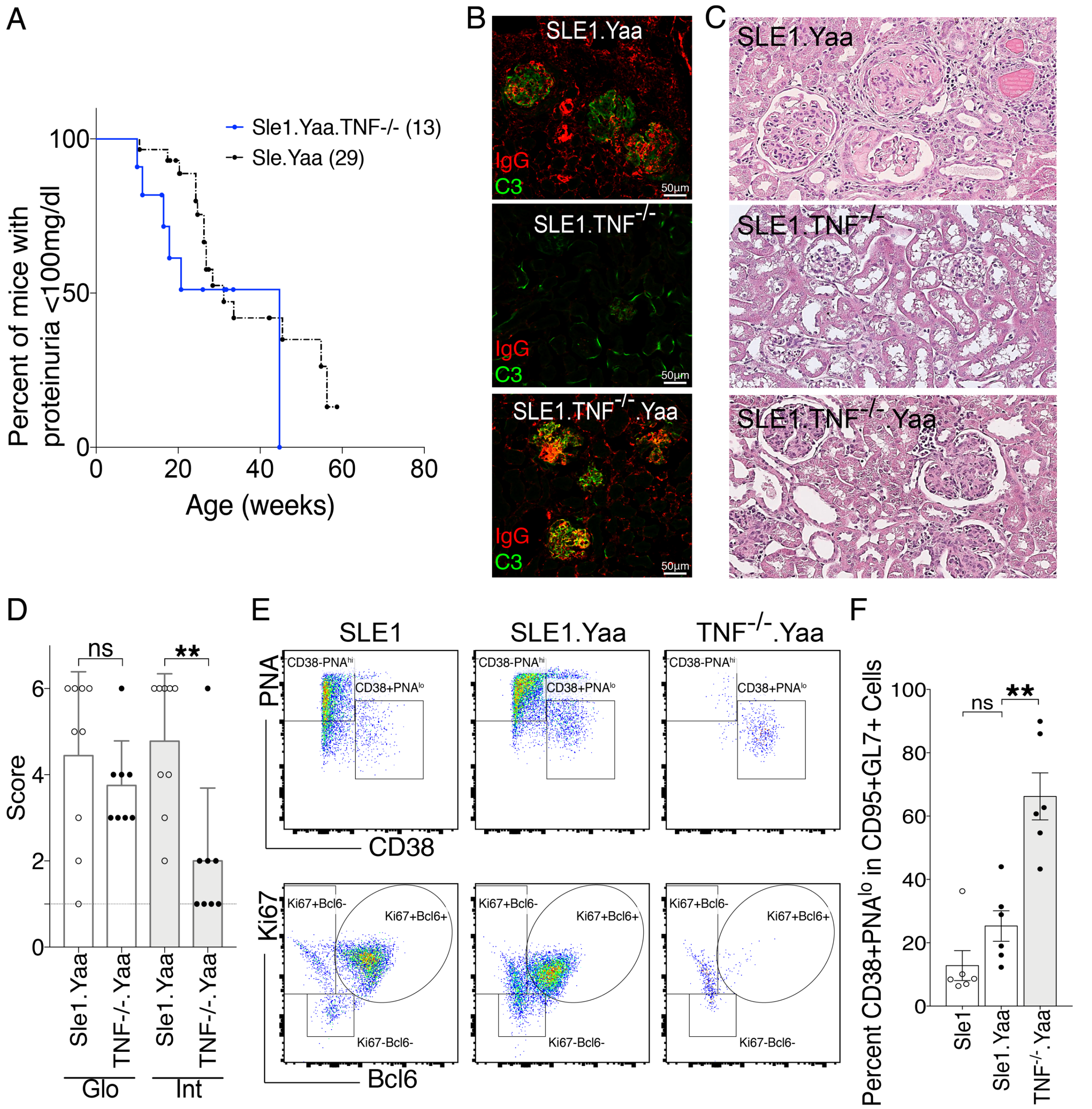

Supplementary Figure 5. Extrafollicular response is associated with clinical disease in Sle1.Yaa.TNF$^{-/-}$ mice. **A.** Percent of mice with proteinuria of <100mg/dl as they age. **B.** IgG and C3 deposition in glomeruli of male Sle1.Yaa and Sle1.Yaa.TNF$^{-/-}$ mice but not in male Sle1.TNF$^{-/-}$ mice. **C.** Representative H&E images show glomerulonephritis and glomerular hypertrophy in > 6-month-old male Sle1.Yaa and Sle1.Yaa.TNF$^{-/-}$ mice but not in male Sle1.TNF$^{-/-}$ mice. **D.** Bar graphs showed summary

of Glomerular (Glo) and interstitial (Int) scores from renal H&E stains. Dotted line indicates maximum values in young Sle1 controls. **E.** Representative plots show PNA, CD38, Ki67 and Bcl6 expression in splenic CD95$^+$GL7$^+$ B cells. **F.** Percent of PNA^{lo}CD38$^+$ cells in CD19$^+$CD95$^+$GL7$^+$ cells in > 6-month-old male Sle1.Yaa, Sle1.TNF$^{-/-}$ and Sle1.Yaa.TNF$^{-/-}$ mice. Dots on bar graphs represent individual mice. ANOVA Kruskal-Wallis with Dunn’s multiple comparisons test, * $p<0.05$: ** $p<0.01$.

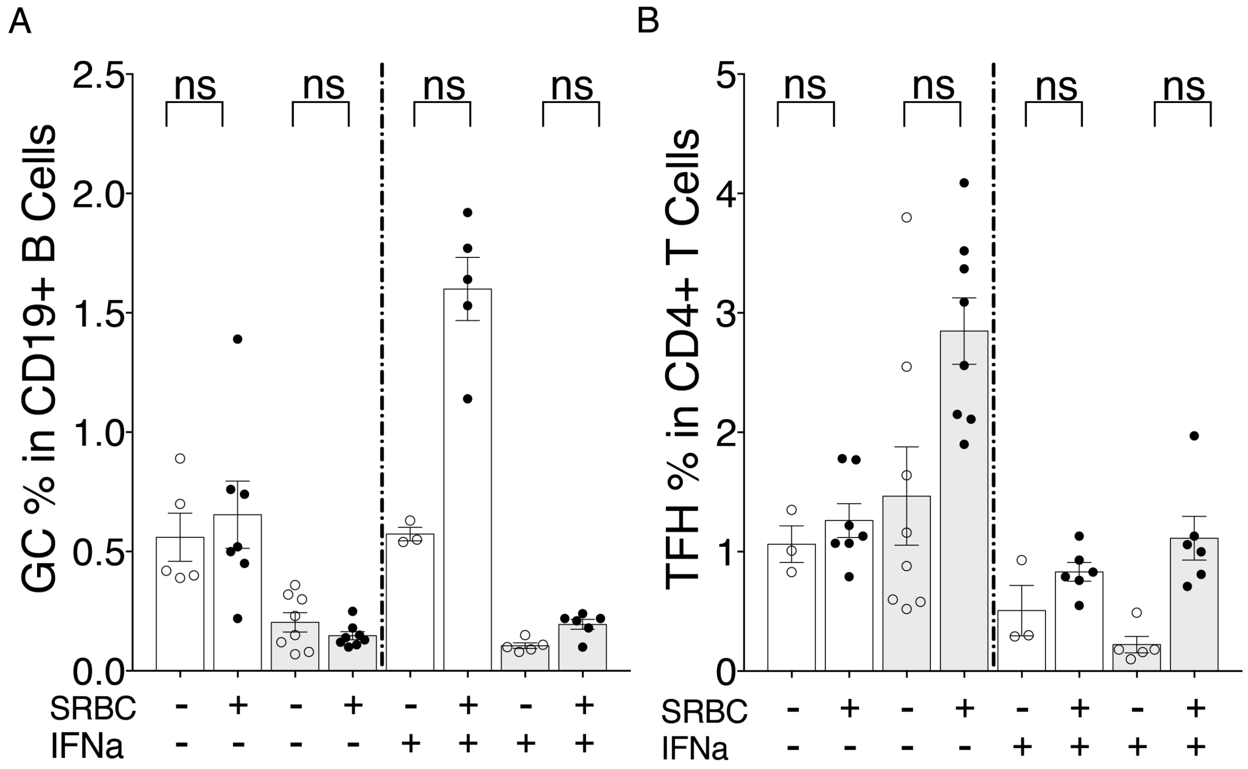

Supplementary Figure 6. T dependent immunization with and without IFNA does not induce GC formation in Sle1.TNF$^{-/-}$ mice. Plots show percent of CD95^{+}GL7^{+} in CD19^{+} B cells (A) and CD44^{+}PSGL1^{lo}PD1^{hi} in CD4^{+} T cells (B) in Sle1 (white bars and Sle1.TNF$^{-/-}$ mice (grey bars) after immunization as indicated. Dots on bar graphs represent individual mice. ANOVA Kruskal-Wallis with Dunn's multiple comparisons test, ns = not significant.

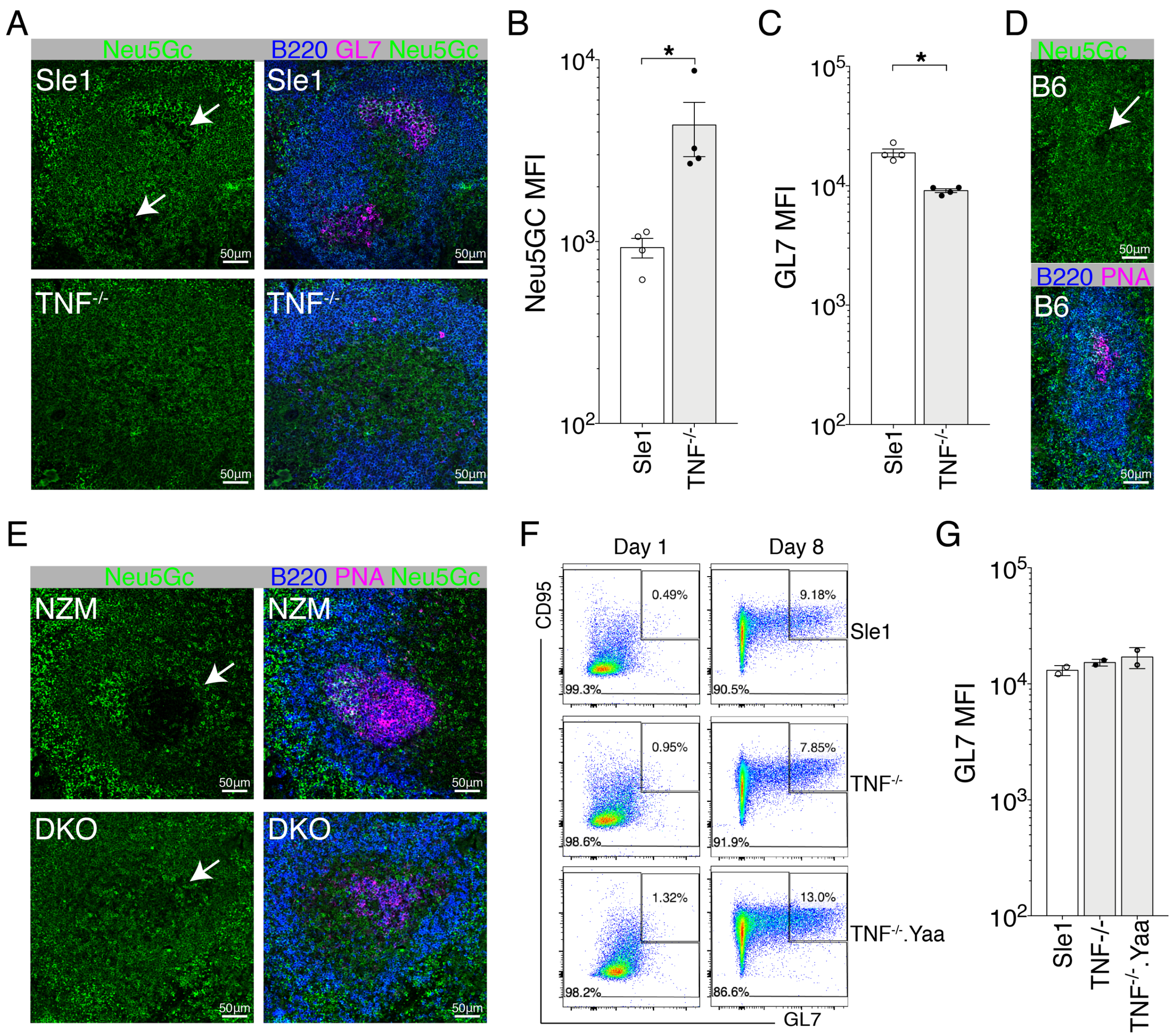

Supplementary Figure 7. Failure to acquire GL7 expression in Sle1.TNF$^{-/-}$ mice is due to sustained expression of Neu5Gc and is B cell extrinsic. A. Immunohistochemistry images (20x) show reduction of Neu5GC expression on GL7^{+} B cells in GC of Sle1 mice (top), but not in Sle1.TNF$^{-/-}$ (bottom) mice. **B-C.** Summary bar graphs of flow analysis shows MFI of Neu5Gc (B) and GL7 (C) on splenic CD95^{+}GL7^{+} B cells. **D.** Immunohistochemistry image (20x) shows reduction of Neu5GC expression on PNA+ B cells in GC of NZM mice (top) that is attenuated in DKO (bottom) mice. **E.** Follicular CD19+ splenic B cells from Sle1.TNF$^{-/-}$ mice were sort purified and placed in

culture with Nojima supporter cells and IL4. Cells were collected at the indicated days and analyzed for CD95 and GL7 expression. Plots show cells gated on singlet live $CD3^-$ $CD19^+$ cells. **F.** Summary bar graph of flow cytometry analysis shows the GL7 MFI of $CD95^+GL7^+$ cells. Dots on bar graphs represent individual mice. Mann-Whitney t-test, * $p<0.05$.
